# Simple and efficient delivery of cell-impermeable organic fluorescent probes into live cells for live-cell superresolution imaging

**DOI:** 10.1038/s41377-019-0188-0

**Published:** 2019-08-14

**Authors:** Meng Zhang, Meihua Li, Wenting Zhang, Yubing Han, Yu-Hui Zhang

**Affiliations:** 1 Britton Chance Center for Biomedical Photonics, Wuhan National Laboratory for Optoelectronics-Huazhong University of Science and Technology, Wuhan, Hubei 430074 China; 2 MoE Key Laboratory for Biomedical Photonics, School of Engineering Sciences, Huazhong University of Science and Technology, Wuhan, Hubei 430074 China

**Keywords:** Super-resolution microscopy, Imaging and sensing

## Abstract

Numerous commercial organic fluorophores with excellent optical properties are precluded from live-cell superresolution imaging due to poor cell permeability. Here, we develop a simple but effective strategy that renders cells permeable to cell-impermeable, organic fluorescent probes by using a novel peptide vehicle, PV-1. By simple coincubation with PV-1, 22 different cell-impermeable, organic fluorescent probes were efficiently delivered into live cells and specifically labeled a variety of organelles. Moreover, PV-1 can simultaneously transfer up to three different probes into live cells. By using PV-1 and these cell-impermeable fluorescent probes, we obtained multicolor, long-term, live-cell superresolution images of various organelles, which allowed us to study the dynamic interactions between them. PV-1, together with these organic fluorescent probes, will greatly broaden the applications of superresolution imaging technology in diverse live-cell studies and opens up a new avenue in the design and application of peptide vehicles.

## Fluorescent probes: Peptide provides permeability

A new peptide that delivers fluorescent probes into live cells, developed by researchers in China, enables real-time imaging of interactions between subcellular structures. Fluorescent probe molecules that attach to specific proteins or structures are vital for high-resolution imaging techniques such as structured illumination microscopy, however most of the best commercial probes can’t easily penetrate cell membranes. Yu-Hui Zhang and co-workers at Huazhong University of Science and Technology in Wuhan found that by incubating probes with a novel peptide called PV-1, they could effectively deliver 22 different probes into cells. PV-1 even enables the delivery of three probe types into a single live cell, labeling structures such as nuclei, actin filaments and mitochondria in different colors. The team could thereby record movies of dynamic interactions, such as the capturing of lysosomes by the endoplasmic reticulum.

## Introduction

Precise imaging of intracellular, subcellular structures and their dynamic processes is crucial for fundamental research in biology and medicine^1–4^. Thanks to recently developed far-field superresolution fluorescence microscopy (e.g., SIM, STED, and PALM/STORM), imaging subcellular structures with a spatial resolution beyond the diffraction limit has been achieved^4–7^. Among these superresolution microscopy methods, structured illumination microscopy (SIM) is distinct in its high imaging speed and low illumination intensities; therefore, it is a standout tool for directly observing the dynamics of subcellular structures in live cells^3,6–9^. However, the application of SIM for characterizing dynamic interactions between subcellular structures remain challenging, as they need fluorescent probes that not only can specifically label various subcellular structures but are also live-cell compatible and possess excellent photostability for long-term observation and broad spectral coverage for multicolor imaging. Genetically encoded fluorescent proteins are live-cell compatible and have been widely used in live-cell fluorescence imaging^1,2,4^. However, organic fluorophores generally offer several important advantages over fluorescent proteins, including smaller sizes, broader spectral coverage, higher fluorescence intensity, and better photostability^1,2,4,10,11^. To date, many cell-permeable organic fluorescent probes have been developed for live cell imaging, and their optical properties (e.g., fluorescence intensity and photostability) are good enough for conventional confocal microscopy^1,2^. However, for long-term multicolor SIM imaging, probes with much better photostability are required^4,8–11^. Over recent decades, numerous small-molecule organic fluorophores (e.g., Alexa Fluor dyes, Atto dyes, Cy dyes, and dyomics dyes) with high fluorescence intensity and excellent photostability have been developed and commercialized, but most of them lack cell permeability without assistance or lose their cell permeability after being conjugated to a recognition unit to achieve specific labeling^10–14^. Therefore, the use of these organic fluorophores to construct fluorescent probes for live-cell SIM applications is severely restricted.

To improve the cellular uptake of organic fluorophores, several strategies have been developed, including chemical modification of fluorophores and the use of physical methods to temporarily disrupt the cell membrane, such as electroporation and nanoinjection^14–16^. However, these methods present several limitations in terms of applicability and efficiency and are also time-consuming and technically demanding. To date, only a few organic fluorophores have successfully been made cell permeable following chemical modification^10,11,14^. Therefore, numerous commercial organic fluorophores with high fluorescence intensity and excellent photostability are precluded from live-cell SIM imaging due to poor cell permeability. On the other hand, characterizing dynamic interactions between subcellular structures in live cells by SIM is difficult to achieve due to the lack of suitable live-cell compatible fluorescent probes.

Herein, we have developed a simple but effective strategy that renders cells permeable to cell-impermeable organic fluorescent probes by using a novel peptide vehicle, PV-1, rather than chemical modification, electroporation, or nanoinjection. By simple coincubation with PV-1, a wide range of cell-impermeable, organic fluorescent probes containing different fluorophores targeting various organelles were efficiently delivered into live cells. Using PV-1 and these cell-impermeable organic fluorescent probes, we obtained multicolor, long-term SIM images of various organelles in live cells, which allowed us to study the dynamic interactions between them. To date and to the best of our knowledge, PV-1 is the first vehicle that can transfer cell-impermeable organic fluorescent probes into live cells with satisfactory efficiency for imaging, requiring only the coincubation of the probes with PV-1.

## Results

### Designing novel peptide vehicles for cell-impermeable organic fluorescent probes

**Fig. 1:**
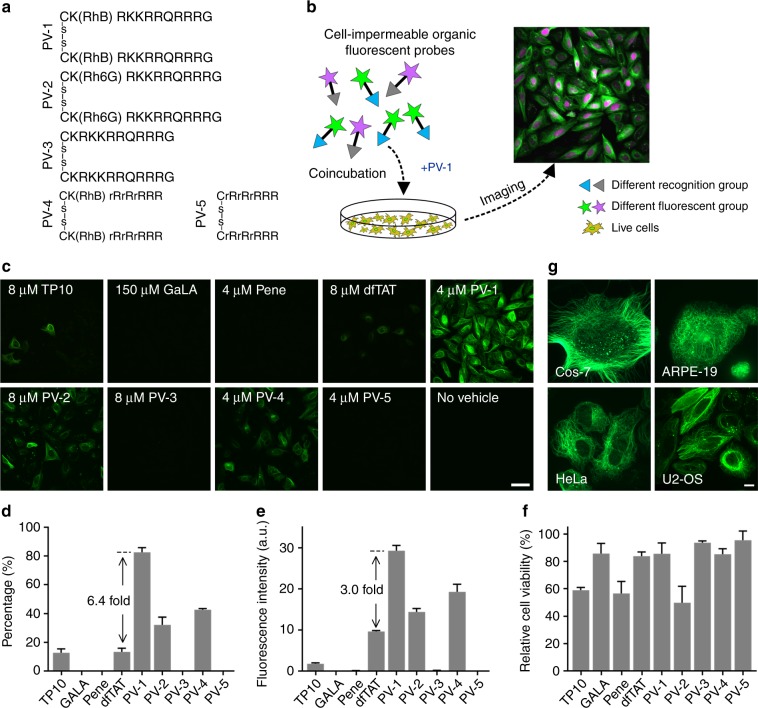
PV-1 efficiently delivers Tubulin-FITC into live cells. **a** The chemical structures of the peptide vehicles. r, d-Arginine; -s-s-, a disulfide bond; RhB, rhodamine B; Rh6G, a rhodamine derivative (fig. S1). **b** A schematic of the intracellular delivery of PV-1. **c** Confocal microscopy images of live U-2 OS cells after a 1-h coincubation with Tubulin-FITC (5 μM) and the indicated peptide vehicles at the indicated optimal concentrations for probe delivery. Scale bar: 50 μm. **d** Percentages of live cells labeled by Tubulin-FITC after a 1-h coincubation with the indicated peptide vehicles (mean + s.d., *n* = 1000 cells from triplicate experiments). **e** The mean fluorescence intensity of Tubulin-FITC inside one cell after a 1-h coincubation with Tubulin-FITC (5 μM) and the indicated peptide vehicles (mean + s.d., *n* = 1,000 cells from triplicate experiments), a.u.: arbitrary units. **f** U-2 OS relative cell viability after a 1-h incubation with the indicated peptide vehicles. The error bars represent the standard deviations of triplicate experiments. The concentrations of the peptide vehicles used in (**d**–**f**) were the same as those used in (**c**). **g** Confocal microscopy images of various live cells after a 1-h coincubation with Tubulin-FITC (5 μM) and PV-1 (4 μM). Scale bar: 10 μm

We first synthesized nine peptide vehicles; among them, PV-1, PV-2, PV-3, PV-4, and PV-5 were novel peptide vehicles designed by us. All of them are disulfide-bonded dimers of short peptides but contain different peptide sequences or fluorophores (Fig. 1a). TP10, GALA, Penetratin (Pene), and dfTAT were selected from previous reports because they all achieved efficient cytosolic delivery of proteins or nucleic acids by simple coincubation (fig. S1 and notes S1–2)^17–20^. However, whether these existing peptide vehicles can be used to deliver cell-impermeable organic fluorescent probes into live cells is still unknown because the sizes and structures of organic fluorescent probes are much different from those of protein or nucleic acids and because their delivery capability greatly depends on the chemical structures of their cargoes^17–19,21,22^. We also synthesized Tubulin-FITC, an organic fluorescent probe containing a docetaxel scaffold that selectively binds to microtubules^23^ and a cell-impermeable dye, FITC (fluorescein isothiocyanate) (table S1).

Live cells were coincubated with Tubulin-FITC and the abovementioned peptide vehicles (Fig. 1b) at different concentrations for 1 h and then imaged by spinning disk confocal microscopy. Our results showed that it was difficult for Tubulin-FITC to enter live cells on its own (Fig. 1c). After coincubation of cells with Tubulin-FITC and GALA, Pene, PV-3, or PV-5, almost no fluorescence from Tubulin-FITC was observed inside cells (Fig. 1c and fig. S2). After coincubation of cells with Tubulin-FITC and TP10 or dfTAT, a weak fluorescence signal from Tubulin-FITC was detected, but not enough to produce clear images (Fig. 1c and fig. S2). Increasing concentrations of these peptide vehicles had little effect on their delivery efficiency (fig. S2), and obvious cytotoxicity was observed when higher concentrations were used (data not shown). By contrast, following coincubation of cells with PV-1 and Tubulin-FITC, approximately 82% of cells were labeled by Tubulin-FITC, and a strong fluorescence intensity of Tubulin-FITC inside cells and specific labeling of microtubules were clearly observed, suggesting an excellent delivery efficacy of PV-1 for Tubulin-FITC (Fig. 1c, d, e). Different peptide sequences, such as TP10, GALA, Pene, PV-1, and PV-4, resulted in significant differences in deliver efficacy (Fig. 1c, d, e), suggesting that the chemical structure of the peptide clearly affects the delivery efficiency, which is consistent with previous reports^21^. Compared with dfTAT, PV-1 contains a different fluorophore, rhodamine B (RhB), which is covalently conjugated to the peptide segment via the ortho-carboxyl group, instead of tetramethylrhodamine (TMR) via the meta-carboxyl group in dfTAT, resulting in a 6-fold increase in the labeling percentage and a threefold increase in the mean fluorescence intensity (Fig. 1d, e). For PV-1 and PV-2, which contain the same peptide sequence, RhB and Rh6G, respectively are conjugated to the peptide segment via the ortho-carboxyl group, which can promote the formation of the lactone isomer^24–26^. The delivery efficacy of PV-1 was much better than that of PV-2 (Fig. 1d, e). These results suggest that the chemical structure of the fluorophore in the vehicle clearly affects the delivery efficiency, whereas in the previous report^17,27^, TMR was only used as a marker for fluorescence imaging. PV-1 and PV-4 have the same peptide sequences as PV-3 and PV-5, respectively, but contain additional RhB. The delivery efficacy of PV-1 and PV-4 were much better than that of PV-3, PV-5, or a mixture of RhB and PV-3, suggesting that the covalent conjugation of RhB plays an essential role in the delivery function of these peptide vehicles (Fig. 1c–e, and figs. S2–3). We also incubated RhB or TMR with Tubulin-FITC. In both cases, no obvious fluorescence of Tubulin-FITC was found inside cells, indicating that the peptide segment is important for the delivery function (fig. S3). It is worth noting that after conjugation with the peptide segment via the ortho-carboxyl group, the fluorescence intensity of RhB in PV-1 decreased dramatically, and almost no clear fluorescence signal of RhB was detected inside the cells (fig. S3, EX: 561 nm) due to the formation of a nonfluorescent “closed” lactone isomer^24–26^, thus minimizing the fluorescence interference from the vehicle to the benefit of multicolor imaging applications. By contrast, dfTAT, when excited by a 561 nm laser, emitted a strong fluorescence signal assigned to TMR after it entered live cells (fig. S3).

Then, we measured cell viability to test the cytotoxicity of these peptide vehicles. Among these peptide vehicles, TP10, Pene, and PV-2 had relatively high cytotoxicity (Fig. 1f). The viability of cells treated with PV-1 at the optimal concentration for probe delivery exceeded 85%, highlighting its low cytotoxicity (Fig. 1f). Therefore, in terms of the delivery efficacy, cytotoxicity and fluorescence background, PV-1 was the optimal candidate among all of the peptide vehicles tested for delivering cell-impermeable organic probes. In addition, PV-1 maintained its excellent delivery capability in various cell lines, including Cos-7, ARPE-19, HeLa, and U-2 OS (Fig. 1g).

To explore the cellular uptake mechanism involved in the delivery of PV-1, we first decreased the incubation temperature to 4 °C, a condition that typically inhibits endocytosis^21^. After a 1-h coincubation with PV-1 and Tubulin-FITC at 4 °C, very weak fluorescence was detected inside live cells, suggesting that endocytosis plays an essential role in the delivery of PV-1 (fig. S4). We then used three inhibitors (filipin, chlorpromazine (CPZ), and 5-(N-ethyl-N-isopropyl) amiloride (EIPA)) of endocytosis to block individual endocytic pathways^21,28^. The results showed that filipin (an inhibitor of caveolae-mediated endocytosis) and CPZ (an inhibitor of clathrin-mediated endocytosis) had little effect on the delivery of PV-1, and EIPA (an inhibitor of macropinocytosis) significantly inhibited the delivery of PV-1, suggesting that micropinocytosis plays an essential role in the efficient delivery of PV-1 (fig. S4). Interestingly, after coincubation with PV-1, EGFP (enhanced green fluorescent protein) was also delivered into live cells, implying that PV-1-mediated transport is not selective for proteins and small-molecule compounds (fig. S5). The presence of FBS (fetal bovine serum) in the cell incubation medium significantly reduced the delivery efficiency of PV-1 (fig. S6), probably because positively charged PV-1 binds to negatively charged FBS^17,29^.

### PV-1 delivers organic probes containing different fluorophores and recognition units

**Fig. 2:**
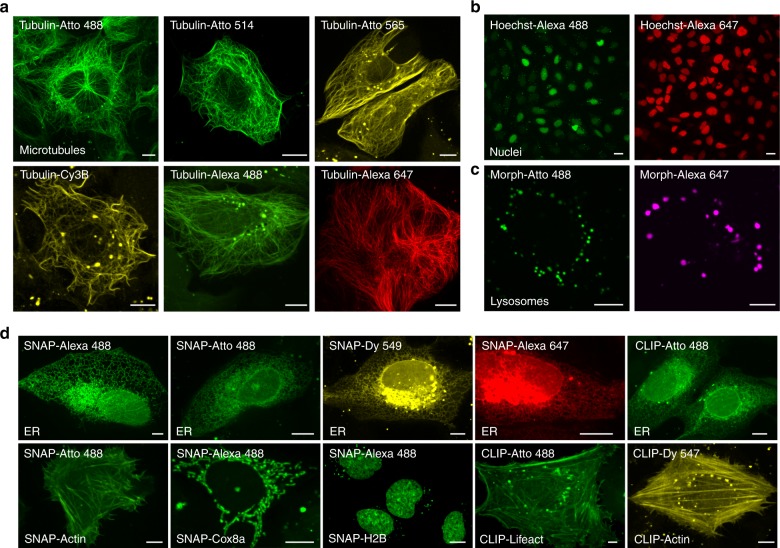
PV-1 efficiently delivers diverse cell-impermeable organic fluorescent probes into live cells. **a** Confocal microscopy images of live U-2 OS cells after a 1-h coincubation with PV-1 (4 μM) and the indicated probes for microtubules with different colors (5 μM). Scale bars: 10 μm. **b** Confocal microscopy images of live U-2 OS cells after a 1-h coincubation with PV-1 (4 μM) and Hoechst-Alexa 488 or Hoechst-Alexa 647 (3 μM). Scale bars: 20 μm. **c** Confocal microscopy images of live U-2 OS cells after a 1-h coincubation with PV-1 (4 μM) and Morph-Atto 488 or Morph-Alexa 647 (5 μM). Scale bars: 5 μm. The labeled organelles are indicated in the bottom left of the first images. **d** Confocal microscopy images of live U-2 OS cells expressing SNAP-Sec61β (top row, four first images), CLIP-Sec61β (top row, last image), SNAP-Actin, SNAP-Cox8a, SNAP-H2B, CLIP-Lifeact or CLIP-Actin after a 1-h coincubation with PV-1 (4 μM) and the indicated SNAP or CLIP probes (5 μM). Scale bars: 5 μm

Next, we investigated whether PV-1 could deliver cell-impermeable organic probes other than Tubulin-FITC containing different organic fluorophores or recognition units into live cells to achieve specific labeling by simple coincubation. A series of cell-impermeable fluorescent probes (15 probes in total) were synthesized and tested: six probes for microtubules (i.e., Tubulin-Atto 488, Tubulin-Atto 514, Tubulin-Atto 565, Tubulin-Cy3B, Tubulin-Alexa 488, and Tubulin-Alexa 647), four probes for nuclei (i.e., Hoechst-Alexa 488, Hoechst-Alexa 647, Hoechst-Cy3B, and Hoechst-Atto 514), and five probes for lysosomes (i.e., Morph-Alexa 488, Morph-Alexa 647, Morph-Cy3B, Morph-Atto 514, and Morph-Atto 488) (table S1 and notes S3-7). Our results showed that the cellular uptake of all of these probes on their own was poor (fig. S7). However, after co-incubation with PV-1, each of these probes was efficiently delivered into live cells and specifically labeled the respective organelles (Fig. 2a–c, figs. S8-10). There were some off-target punctate signals detected, as shown in Fig. 2a. Our colocalization studies with Tubulin-Alexa 647 (fig. S11) revealed that these punctate vesicles colocalized with LysoTracker Green, a probe that labels lysosomes, but not with Rab5b-GFP, a marker protein of early endosomes^30^, implying that only a relatively small amount of the probes was trapped inside lysosomes, while most reached the cytosol and labeled the target organelles. The PV-1 delivery method is limited in labeling punctate structures except lysosomes unless off-target punctate signals can be excluded by colocalization with lysosomal trackers. In sum, PV-1 can deliver cell-impermeable organic probes containing different fluorophores and recognition units into live cells, implying a wide range of promising applications of PV-1 in live-cell studies.

### PV-1 delivers commercial cell-impermeable SNAP/CLIP probes

SNAP-tag/CLIP-tag labeling technologies provide simple, robust, and versatile approaches for specific labeling of fusion proteins with organic fluorescent probes; therefore, they are powerful tools for imaging proteins and can be used in a wide range of experimental applications^31–34^. A series of SNAP probes (i.e., SNAP-TMR, SNAP-SiR647, SNAP-Atto 488, SNAP-Alexa 488, SNAP-Dy 549, and SNAP-Alexa 647) and CLIP probes (i.e., CLIP-TMR, CLIP-Atto 488, and CLIP-Dy 547) bearing fluorophores with excellent optical properties have been commercialized (table S1); however, most of them (except SNAP-TMR, SNAP-SiR647, and CLIP-TMR) are cell-impermeable and thus are primarily restricted to labeling proteins on the surface of live cells. SNAP-TMR, SNAP-SiR647, and CLIP-TMR are cell-permeable but they have similar excitation wavelengths, which limits their applications in multicolor imaging.

To address this issue, we investigated whether PV-1 could be used to deliver these commercial cell-impermeable SNAP/CLIP probes into live cells. U-2 OS cells expressing SNAP-Sec61β decorating the endoplasmic reticulum (ER) were incubated with SNAP probes in the absence or presence of PV-1 for 1 h. Remarkably, after coincubation with PV-1, all of the SNAP probes were efficiently translocated into live cells, and specific labeling of the ER was clearly observed (Fig. 2d). By contrast, no obvious fluorescence was detected inside live cells for any of the SNAP and CLIP probes in the absence of PV-1 (fig. S7). Using PV-1 and the SNAP or CLIP probes, we also successfully labeled F-actin, mitochondria, and nuclei in live cells expressing SNAP-Actin, SNAP-Lifeact, CLIP-Actin, CLIP-Lifeact, SNAP-Cox8a, or SNAP-H2B (Fig. 2d and fig. S12). To investigate the labeling specificity, we performed colocalization studies using the anti-actin antibodies Alexa 568, Hoechst 33342, BFP-KDEL^8,35^, and MitoTracker Deep Red as the standard markers for actin, nuclei, the ER, and mitochondria, respectively. The results showed that these SNAP and CLIP probes can label the desired targets with high specificity (fig. S13). Considering the universality of SNAP-tag/CLIP-tag technologies for protein labeling, efficient delivery of these commercial cell-impermeable SNAP/CLIP probes by PV-1 into live cells will greatly benefit the imaging of intracellular proteins in live cells for a wide range of applications.

### PV-1 simultaneously delivers up to three cell-impermeable organic probes

**Fig. 3:**
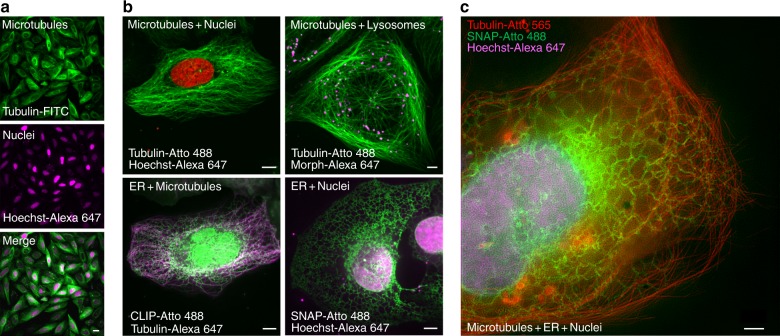
PV-1 simultaneously delivers up to three cell-impermeable organic fluorescent probes into live cells. **a** Confocal microscopy images of live U-2 OS cells after a 1-h coincubation with PV-1 (4 μM), Tubulin-FITC (5 μM), and Hoechst-Alexa 647 (3 μM). Scale bar: 50 μm. **b** Confocal microscopy images of live U-2 OS cells wild-type (top) or expressing CLIP-Sec61β (bottom left) or SNAP-Sec61β (bottom right) after a 1-h coincubation with PV-1 (4 μM) and the two indicated probes (Hoechst-Alexa 647: 3 μM; the others: 5 μM). Scale bars: 10 μm. **c** A triple-color SIM image of live U-2 OS cells expressing SNAP-Sec61β after a 1-h coincubation with PV-1 (4 μM), Tubulin-Atto 565 (5 μM), SNAP-Atto 488 (5 μM), and Hoechst-Alexa 647 (3 μM). Scale bar: 2 μm. The labeled organelles are indicated in the panels

Multicolor fluorescence imaging has greatly advanced our understanding of the interactions among biomolecules in live cells. To determine whether PV-1 could be used to deliver more than one organic probe into live cells simultaneously for multicolor imaging applications, we incubated PV-1 with Tubulin-FITC and Hoechst-Alexa 647 at the same time. Notably, both Tubulin-FITC and Hoechst-Alexa 647 were efficiently delivered into live cells, and microtubules and nuclei were simultaneously visualized in the same live cells (Fig. 3a). Under normal circumstances, more than 90% of live cells are in interphase; therefore, most of the labeled cells are interphase cells (Fig. 3a), but cells in other stages of the cell cycle (e.g., prophase, metaphase, anaphase, and telophase) can also be labeled and observed (fig. S14). We also successfully labeled microtubules and nuclei, the ER and nuclei, or microtubules and the ER simultaneously by delivering two corresponding probes with PV-1 into live cells (Fig. 3b and fig. S15) Most notably, we simultaneously stained microtubules, nuclei, and the ER in the same live cells by concurrent coincubation of Tubulin-Atto 565, Hoechst-Alexa 647, and SNAP-Surface Atto 488 with PV-1, and we obtained triple-color live-cell SIM superresolution images of these three organelles (Fig. 3c). These results demonstrate that PV-1 can be used to simultaneously deliver two or more organic probes into live cells for multicolor imaging applications.

### Live-cell SIM imaging reveals the dynamic interactions between organelles

**Fig. 4:**
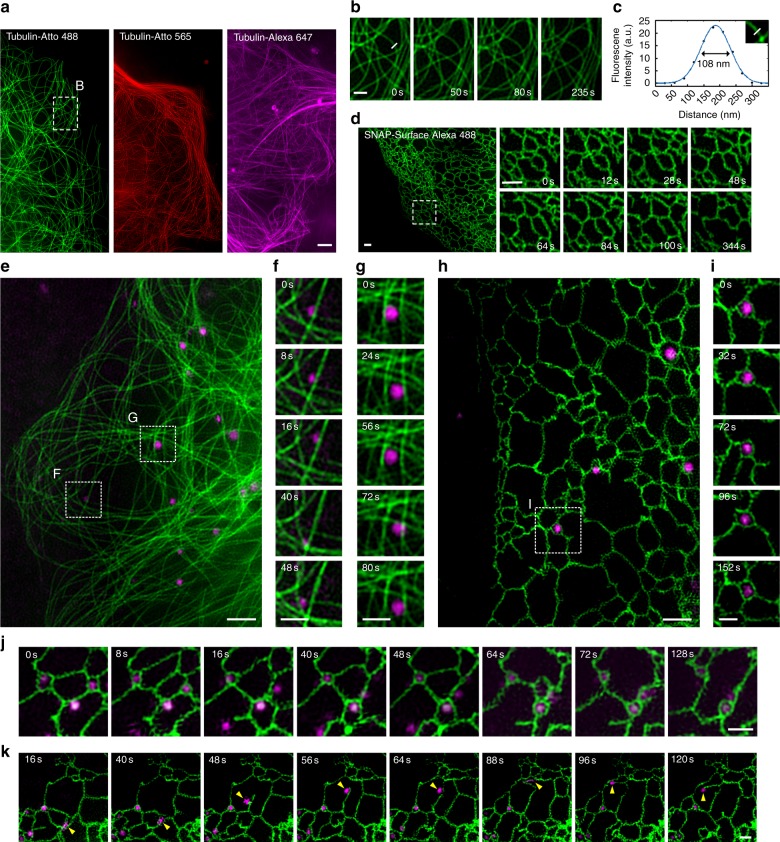
Live-cell SIM imaging reveals the dynamics of organelles. **a** SIM images of live U-2 OS cells after a 1-h coincubation with PV-1 (4 μM) and Tubulin-Atto 488, Tubulin-Atto 565, or Tubulin-Alexa 647 (5 μM). Additional frames are shown in movie S1. **b** Enlarged time-lapse images of the boxed region in (**a**). Representative images of consecutive SIM frames are displayed, and additional frames are shown in movie S2. **c** The cross-sectional profile of microtubules in (**b**) with full-width at half-maximum (FWHM) of 108 nm. **d** An SIM image of live U-2 OS cells expressing SNAP-Sec61β after a 1-h coincubation with PV-1 (4 μM) and SNAP-Alexa 488 (5 μM), and enlarged time-lapse images of the boxed region. Representative images of consecutive SIM frames are displayed, and additional frames are shown in movies S3 and S4. **e** A dual-color SIM image of live U-2 OS cells after a 1-h coincubation with PV-1 (4 μM), Tubulin-Atto 488 (5 μM), and Morph-Alexa 647 (3 μM). Additional frames are shown in movie S5. **f**, **g** Enlarged time-lapse images of the boxed regions in (**e**). Representative images of consecutive SIM frames are displayed, and additional frames are shown in movies S6 and S7. **h** A dual-color SIM image of live U-2 OS cells expressing SNAP-Sec61β after a 1-h coincubation with PV-1 (4 μM), SNAP-Alexa 488 (5 μM), and Morph-Alexa 647 (3 μM). Additional frames are shown in movie S8. **i** Enlarged time-lapse images of the boxed region in (**h**) reveal dynamic interactions between lysosomes and the ER. Representative images of consecutive SIM frames are displayed, and additional frames are shown in movie S9. **j**, **k** Representative time-lapse SIM images reveal different types of dynamic interactions between lysosomes and the ER in live U-2 OS cells expressing SNAP-Sec61β after a 1-h coincubation with PV-1 (4 μM), SNAP-Alexa 488 (5 μM), and Morph-Alexa 647 (3 μM). Additional frames are shown in movies S10 and S11. Scale bars: **a**, **e**, and **h**, 2 μm; **b**, **d**, **f**, **g**, **i**, **j** and **k**, 1 μm

PV-1, together with these organic fluorescent probes (table S1), provided more live-cell compatible fluorescent probes for SIM. By using PV-1 and these cell-impermeable fluorescent probes, we obtained SIM images of microtubules, the ER, and nuclei in live cells (Fig. 4a–d and fig. S16). Notably, microtubule and the ER dynamics were continuously recorded in live cells with SIM without obvious photobleaching for up to ∼4 or 6 min (Fig. 4b, d, and movie S1-4). Moreover, we were able to record the dynamic interactions between lysosomes and microtubules (Fig. 3e–g and movie S5–7) and between lysosomes and the ER (Fig. 4h–k and movie S8–11) in live cells over a time course of ∼3 min with dual-color SIM. Our imaging captured the trafficking of a lysosome along the microtubules (Fig. 4f and movie S6) and a lysosome trapped within the microtubule network (Fig. 4g and movie S7). Most interestingly, these consecutive SIM superresolution images allowed us to identify three types of interactions between lysosomes and the ER: (1) lysosomes were tightly surrounded and trapped within the ER network (Fig. 4i and movie S9); (2) ER tubules captured a lysosome by gradually narrowing the tubule ring (Fig. 4j and movie S10); and (3) a lysosome was bound to the end of an ER tubule and moved together with the tubule (Fig. 4k and movie S11).

## Discussion

In summary, we have developed a simple but effective strategy for efficient cytosolic delivery of cell-impermeable organic fluorescent probes by using a novel peptide vehicle, PV-1. By simple coincubation, we showed that PV-1 can efficiently deliver 22 different cell-impermeable organic fluorescent probes into live cells, specifically labeling various intracellular organelles, including microtubules, nuclei, mitochondria, lysosomes, the ER, and F-actin.

Numerous peptide vehicles (more than 100) have been widely used to deliver cell-impermeable compounds (e.g., fluorophores, drugs, and biomacromolecules) into live cells^21^. The structures of these peptide vehicles differ greatly in amino acid composition, three-dimensional structure, and polarity (e.g., cationic, anionic, and neutral sequences). Their delivery mechanisms also vary greatly according to the chemical structure of their cargoes^21^. Thus far, by simple coincubation, peptide vehicles can only efficiently deliver nucleic acids and proteins into live cells. The peptide vehicles used to deliver nucleic acids are usually cationic peptides, which transfer nucleic acids through electron interactions between them^19,21^. Meanwhile, to achieve the cytosolic delivery of proteins, peptide vehicles need to disrupt endosomes to induce effective endoplasmic leakage after the endocytic uptake of proteins^17^. Because the chemical structures and polarities of small-molecule organic fluorescent probes are significantly different from nucleic acids and proteins, those peptide vehicles (e.g., TP10, GALA, Pene, and dfTAT) that achieved efficient cytosolic delivery of proteins or nucleic acids lost their excellent delivery capability for small-molecule organic fluorescent probes (Fig. 1c). To date, no effective cytosolic delivery of organic fluorescent probes by peptide vehicles via simple coincubation has been reported. The general approach in previous reports to deliver cell-impermeable small molecules with peptide vehicles involves connecting these small molecules with peptide vehicles through covalent bonds, which generally involves complex and time-consuming synthesis processes^9,18,21,22^. To the best of our knowledge, this is the first time that a peptide vehicle has been shown to transfer cell-impermeable organic fluorescent probes into live cells with satisfactory efficiency for imaging by simple coincubation. To date, it is still difficult to predict what kind of peptide sequence can efficiently transport small-molecule organic fluorescent probes by simple coincubation or to summarize the relationship between the peptide sequence and the delivery efficiency. However, our results open up a new possibility for the design and applications of peptide vehicles.

The cell-impermeable organic probes that we tested and were successfully delivered by PV-1 display diverse chemical structures and polarities (table S1), highlighting potentially broad uses of PV-1 as a vehicle. Predicting the delivery efficacy of PV-1 for other untested fluorescent probes is difficult because the delivery capability of these kind of transports greatly depends on their cargoes^17–21^; however, most organic fluorophores with excellent optical properties that are suitable for long-term SIM imaging are rhodamine derivatives (e.g., Alexa 488, Atto 488, and Atto 565) or anthocyanin derivatives (e.g., Cy 3B, Alexa 647, and Dy 549), and it is likely that the excellent delivery capability of PV-1 is maintained for both types of these derivatives. The ability of PV-1 to deliver these cell-impermeable organic fluorophores into live cells will greatly expand the applications of these fluorophores in the field of live cell research and can also reduce the contradiction between cell permeability and optical properties in the process of developing new organic fluorophores.

Among the probes tested, we successfully delivered the commercially available, cell-impermeable SNAP and CLIP probes, which are used together with the robust and versatile SNAP- and CLIP-tag technologies to label specific proteins. Therefore, the efficient delivery of SNAP and CLIP probes by PV-1 could advance the imaging of intracellular proteins in live cells across a wide range of applications. Of particular importance is our finding that PV-1 can simultaneously deliver up to three different probes into live cells, providing a new approach for labeling intracellular targets for multicolor live-cell imaging applications. Using PV-1 and these cell-impermeable probes, multicolor consecutive SIM images of various organelles in live cells were obtained. This allowed us to identify three different types of interactions between lysosomes and the ER. We anticipate that PV-1, together with these organic fluorescent probes, will lay a solid foundation for the application of SIM in diverse live-cell studies, such as protein trafficking, organelle dynamics, and protein-protein interactions.

## Materials and methods

### Chemical synthesis

The methods for the chemical synthesis and characterization of the probes and peptide vehicles can be found in note S1-7.

### Recombinant plasmids

The SNAP-Sec61β vector was generously provided by Prof. Liangyi Chen (Peking University). The SNAP-Cox8a vector was purchased from New England Biolabs. SNAP-actin was generated by substituting F-actin for Sec61β in the SNAP-Sec61β vector. F-actin fragment with BglII and EcoRI sites was first amplified from pAcGFP1-actin using primers (5′-TGG GCT GGG TAG ATC TAT GGA TGA TGA TAT-3; 5′-GTC GAC TGC AGA ATT CCT AGA AGC ATT TGC G-3′). Then the F-actin fragment was inserted into the corresponding site of the linearized SNAP-Sec61β vector. SNAP-Lifeact was generated by substituting Lifeact for Sec61β in the SNAP-Sec61β vector. Oligonucleotides with the sequences 5′-GAT CTA TGG GCG TGG CCG ACT TGA TCA AGA AGT TCG AGT CCA TCT CCA AGG AGG AGT GAG- 3′ and 3′-ATA CCC GCA CCG GCT GAA CTA GTT CTT CAA GCT CAG GTA GAG GTT CCT CCT CAC TCT TAA- 5′, which encode the Lifeact sequence with BglII and EcoRI sites, were annealed to generate dsDNA. The SNAP-Sec61β vector was cut with BglII and EcoRI and then ligated with Lifeact dsDNA. SNAP-H2B was generated by substituting H2B for Sec61β in the SNAP-Sec61β vector. The sequence of H2B that contains the BglII and EcoRI sites was acquired by chemical synthesis (Tsinke). The fragment of H2B was inserted into the corresponding sites of the linearized SNAP-Sec61β vector. The CLIP-Sec61β and CLIP-Lifeact vectors were generated by substituting CLIP for SNAP in the SNAP-Sec61β vector or the SNAP-Lifeact vector, respectively. Full-length CLIP-Tag, which contains NheI and BglII sites were acquired by chemical synthesis (Tsinke). The fragment of CLIP was then inserted into the corresponding sites of the linearized SNAP-Sec61β vector or the SNAP-Lifeact vector. Tubulin-mCherry was generated by substituting mCherry for SNAP in the SNAP-Tubulin vector. mCherry cDNA was first amplified from Dyn2-pmCherryN1 using primers (5′-TGG GCT GGG TGC TAG CGC CAC CAT GGT GAG CAA GG-3′; 5′-GTC GAC TGC AAG ATC TCT TGT ACA GCT CGT CCA TGC C-3′). Dyn2-pmCherryN1 was a gift from Christien Merrifield (Addgene plasmid #27689; http://n2t.net/addgene:27689; RRID: Addgene_27689). All primers were synthesized by Tsinke Biotechnology Co., Ltd. (Wuhan, China). GFP-Rab5B was a gift from Gia Voeltz (Addgene plasmid # 61802; http://n2t.net/addgene:61802; RRID: Addgene_61802). BFP-KDEL was a gift from Gia Voeltz (Addgene plasmid #49150; http://n2t.net/addgene:49150; RRID: Addgene_49150) We confirmed the fidelity of the above plasmids by sequencing.

### Cell lines

HeLa cells were cultured in 10% (vol/vol) FBS (fetal bovine serum; Gibco) DMEM medium (Gibco); Cos-7 and AREP-19 cells were cultured in 10% FBS RPMI 1640 medium (Gibco); U-2 OS cells were cultured in 10% FBS McCoy’s 5A medium (Gibco). All the cell culture media were contained 1% antibiotic-antimycotic (Gibco).

### Transfection

About 1 × 10^5^ U-2 OS cells per well were seeded into a 24-well plate. After 24–36 h, transfections were performed in OPTI-MEM medium (Invitrogen) with 1 μl of Lipofectamine 3000 (Invitrogen) and 500 ng of the indicated plasmids. After 1 day, cells were digested with trypsin-EDTA (0.25%) (Gibco) and then seeded into cell culture dishes (15 mm diameter, NEST) at a density of 8 × 10^4^ per well. Before incubation with PV-1 and the indicated probes, the transfected cells were cultured for 12–24 h.

### Delivery of Tubulin-FITC

About 8 × 10^4^ cells per dish were seeded into 15 mm cell culture dishes (NEST). After 1 day incubation, cells were 70–90% confluent, and all the dishes were washed with PBS (phosphate buffered saline, Gibco). A solution of Tubulin-FITC (5 μM, 100 μl) and the indicated peptide vehicle at the indicated concentrations in fresh medium (without FBS) was then added, and cells were cultured with 5% CO_2_ at 37 °C and for 1 h. After that, all the dishes were washed with PBS and then incubated with Hoechst 33258 (5 μM, 100 μl) for 30 min. Next, remove the supernatant, and all the dishes were washed with PBS and then incubated in 10% FBS cell culture medium with for 1 h. Before imaging, using OPTI-MEM medium (200 μl, Invitrogen) replacing the cell culture medium. Hoechst 33342 as a marker for nuclei was used to count cells, and ImageJ (National Institutes of Health) was used to count the cells that were labeled with Tubulin-FITC (green) and total cells (blue). The ratio of green to blue can determine the percentage of live cells labeled by each peptide-vehicle.

### Live-cell labeling

8 × 10^4^ cells per dish were seeded in 15 mm cell culture dishes. After 1 day incubation, cells were transfected with indicated plasmids or not as described above. After that, a solution of PV-1 (4 μM, 100 μl) and the indicated probe at the indicated concentrations in fresh medium (without FBS) was added, and the cells were cultured with 5% CO_2_ at 37 °C for 1 h. Then remove the supernatant, and all the dishes were washed with PBS and then incubated in 10% FBS cell culture medium with for 1 h.

### Imaging by confocal microscopy

The fluorescence signals were detected using a confocal microscope (Olympus) outfitted with an UltraVIEW VoX 3D live cell imaging system (PerkinElmer) and a sCMOS camera with optional exposure time. The images of Tubulin-Atto 514 and Hoechst-Atto 514 were acquired using an LSM-710 confocal laser scanning microscope (Carl Zeiss). We use ImageJ software to analyze all the fluorescence images.

### Treatment with inhibitors

When treating cells with chlorpromazine or Filipin, cells were first incubated with 10 µg/ml chlorpromazine (Sigma) or 5 µg/ml Filipin (Sigma) in 10% FBS McCoy’s 5 A medium for 30 min. After that, the cells were incubated with 4 μM PV-1 and 5 μM Tubulin-FITC for 1 h. Then cells were washed with PBS and incubated in 10% FBS fresh medium for 1 h.

When treating with EIPA (5-(N-ethyl-N-isopropyl) amiloride), cells were incubated with 100 μM EIPA, 4 μM PV-1, and 5 μM Tubulin-FITC for 1 h. After that, the cells were washed with PBS and incubated in cell culture medium 10% FBS for 1 h.

After the above treatment, cells were imaged by confocal microscopy or analyzed by microcapillary flow cytometer (Guava easyCyte 8HT, EMD Millipore Corporation).

### Cell viability assays

We used MTS (3-(4,5-dimethylthiazol-2-yl)-5-(3-carboxymethoxyphenyl)-2-(4-sulfophenyl)-2H-tetrazolium) assay to evaluate the effects of various peptide vehicles on cell viability. 1 × 10^4^ U-2 OS cells per dish were seeded in a 96-well plate and cultured with 5% CO_2_ at 37 °C for 1 day. Then, all the wells were washed with PBS and incubated with the solution of the peptide vehicles in fresh medium (without FBS) at 37 °C for 1 h. After that, all the wells were washed with fresh medium, and then, added 20 μl of CellTiter 96^®^AQueous One Solution Reagent (Promega) and 80 μl of fresh medium to each well. The 96-well plate was cultured at 37 °C with 5% CO_2_ for 3 h. Finally, we used TECAN GENios Plus ELISA reader (Tecan) to recorded the results.

### Colocalization studies

For punctate vesicle colocalization, one dish of U-2 OS cells expressing Rab5b-GFP was coincubated with 4 μM PV-1 and 5 μM Tubulin-Alexa 647 for 1 h, after that cells were washed with PBS and fresh medium before imaging. The other dish of cells was coincubated with 4 μM PV-1 and 5 μM Tubulin-Alexa 647 for 1 h and then incubated with 500 nM LysoTracker Green (Thermo) for 30 min. Finally, the dish was washed with PBS and fresh medium before imaging.

For microtubule colocalization, U-2 OS cells expressing Tubulin-mCherry were coincubated with 4 μM PV-1 and 5 μM Tubulin-Atto 488 for 1 h. Finally, cells were washed with PBS and fresh medium before imaging.

For nuclear colocalization, one dish of U-2 OS cells was incubated with 4 μM PV-1 and 3 μM Hoechst-Alexa 647 for 1 h. The other dish of U-2 OS cells expressing SNAP-H2B was coincubated with 4 μM PV-1 and 3 μM SNAP-Alexa 488 for 1 h, and then, all dishes were incubated with Hoechst 33342 (10 μg/ml, 100 µl; Thermo) for 15 min and washed with PBS and fresh medium before imaging.

For lysosome colocalization, U-2 OS cells were coincubated with 4 μM PV-1 and 5 μM Morph-Alexa 647 for 1 h and then incubated with LysoTracker Red (Thermo) for 30 min. Finally, cells were washed with PBS and fresh medium before imaging.

For actin colocalization, U-2 OS cells expressing SNAP-actin or CLIP-Lifeact were coincubated with 4 μM PV-1 and 3 μM SNAP-Alexa 488 or 3 μM CLIP-Atto 488 for 1 h and then fixed with 100% methanol, then cells were permeabilized with 0.2% Triton X-100 (Sigma) for 5 min, after that cells were blocked with 3% bovine serum albumin (Sigma) and 0.05% Triton X-100 for 1 h. Finally, cells were incubated at 4 °C with anti-actin antibody Alexa 568 (Abcam, ab208143) at a 1/100 dilution for more than 12 h.

For ER colocalization, U-2 OS cells coexpressing BFP-KDEL and SNAP- Sec61β were coincubated with 4 μM PV-1 and 3 μM SNAP-Alexa 488 for 1 h and then washed with PBS and fresh medium three times before imaging.

For mitochondrial colocalization, U-2 OS cells expressing SNAP-Cox8a were coincubated with 4 μM PV-1 and 3 μM SNAP-Alexa 488 for 1 h and then incubated with MitoTracker Deep Red (50 nM, 100 µl; Thermo) for 30 min and washed with PBS and fresh medium three times before imaging.

All the colocalization results were analyzed by ImageJ software and Volocity 6.3 (PerkinElmer).

### Structured illumination microscopy

Nikon N-SIM microscope outfitted with an apochromat oil immersion objective (100×, 1.49 NA). All the SIM images of living cells were captured and reconstructed using NIS-Elements (Nikon). The images were analyzed with ImageJ, MATLAB software, and NIS-Elements.

## Supplementary information

Supporting Information

video S1

video S2

video S3

video S4

video S5

video S6

video S7

video S8

video S9

video S10

video S11
