# Author Correction: Simple and efficient delivery of cell-impermeable organic fluorescent probes into live cells for live-cell superresolution imaging

**DOI:** 10.1038/s41377-019-0240-0

**Published:** 2020-01-06

**Authors:** Meng Zhang, Meihua Li, Wenting Zhang, Yubing Han, Yu-Hui Zhang

**Affiliations:** 1 Britton Chance Center for Biomedical Photonics, Wuhan National Laboratory for Optoelectronics-Huazhong University of Science and Technology, Wuhan, Hubei 430074 China; 2 MoE Key Laboratory for Biomedical Photonics, School of Engineering Sciences, Huazhong University of Science and Technology, Wuhan, Hubei 430074 China

**Keywords:** Applied optics, Imaging and sensing

**Correction to: Light: Science & Applications**

10.1038/s41377-019-0188-0 published online 14 August 2019

Because dfTAT (*Nat Methods*, 2014, 11(8): 861−867) and 3TAT (*Traffic*, 2018, 19(6): 421−435) have been reported to be able to deliver SNAP-surface 488 into live cells, our claim that “PV-1 is the first vehicle that can transfer cell-impermeable organic fluorescent probes into live cells with satisfactory efficiency for imaging, requiring only the coincubation of the probes with PV-1” is inaccurate. To address this discrepancy, we have made the following corrections to the article on the recommendations of the editor.

1. On page 2, “However, these methods present several limitations in terms of applicability and efficiency and are also time-consuming and technically demanding. To date, only a few organic fluorophores have successfully been made cell permeable following chemical modification^10,11,14^” should be “However, these methods present several limitations in terms of applicability and efficiency and are also time-consuming and technically demanding. Delivering organic fluorophores by non-covalently linked peptide vehicles is an alternative method that is more convenient and cost-efficient^17,36^. However, only a few organic fluorophores have successfully been made cell permeable following chemical modification or simple coincubation with peptide vehicles^10,11,14,17,36^.” to make the statement more accurate.
2. On page 2, “To date and to the best of our knowledge, PV-1 is the first vehicle that can transfer cell-impermeable organic fluorescent probes into live cells with satisfactory efficiency for imaging, requiring only the coincubation of the probes with PV-1.” should be “To date and to the best of our knowledge, PV-1 is the first vehicle that can simultaneously deliver up to three different probes into live cells with satisfactory efficiency for imaging, providing a new approach for labelling intracellular targets for multicolour live-cell imaging applications.” to make the statement more accurate.
3. On page 8, “Thus far, by simple coincubation, peptide vehicles can only efficiently deliver nucleic acids and proteins into live cells” should be “Thus far, by simple coincubation, most peptide vehicles can only efficiently deliver nucleic acids and proteins into live cells, except for dfTAT^17^ and 3TAT^36^.” to make the statement more accurate.
4. On page 8, “To date, no effective cytosolic delivery of organic fluorescent probes by peptide vehicles via simple coincubation has been reported.” should be “The effective cytosolic delivery of a limited number of organic fluorescent probes by peptide vehicles via simple coincubation has been reported^17,36^.” to make the statement more accurate.
5. On page 8, “To the best of our knowledge, this is the first time that a peptide vehicle has been shown to transfer cell-impermeable organic fluorescent probes into live cells with satisfactory efficiency for imaging by simple coincubation.” should be “To the best of our knowledge, this is the first time that a peptide vehicle has been shown to simultaneously transfer up to three different probes into live cells with satisfactory efficiency for imaging by simple coincubation.” to make the statement more accurate.
6. On page 8, “Because the chemical structures and polarities of small-molecule organic fluorescent probes are significantly different from nucleic acids and proteins, those peptide vehicles (e.g., TP10, GALA, Pene, and dfTAT) that achieved efficient cytosolic delivery of proteins or nucleic acids lost their excellent delivery capability for small-molecule organic fluorescent probes (Fig. 1c).” should be “Because the chemical structures and polarities of small-molecule organic fluorescent probes are significantly different from nucleic acids and proteins, most peptide vehicles (e.g., TP10, GALA and Pene) that resulted in the efficient cytosolic delivery of proteins or nucleic acids lost their excellent delivery capability for small-molecule organic fluorescent probes (Fig. 1c and Fig. S2). In addition to delivering proteins, dfTAT has been shown to deliver SNAP-Atto 488 into live cells^17^. However, the live-cell imaging of subcellular structures requires a vehicle not only to deliver probes into live cells but also to deliver them with a sufficient delivery efficiency to obtain adequate signal-to-noise ratios that yield clear images. After coincubation of the cells with dfTAT and the probes (Tubulin-FITC, SNAP-Atto 488, and SNAP-Alexa 488), weak fluorescence signals from these probes were detected inside the cells, but they were insufficient for producing clear images of these subcellular structures (Fig. 1c and Figs. S2, S17). Moreover, the strong fluorescence signal from TMR in dfTAT generated an extremely high background signal (emission wavelength: 580 nm, Figs. S3, S17), which is also a major obstacle for dfTAT as a vehicle for imaging, especially for multicolour imaging. Compared with dfTAT, much stronger probe fluorescence intensities were observed inside the cells after coincubation with PV-1, suggesting an excellent delivery efficacy (Fig. 1c and Figs. S2, S17). Meanwhile, after conjugation with the peptide segment via the ortho-carboxyl group, the fluorescence intensity of RhB in PV-1 decreased dramatically, and almost no clear fluorescence signal from RhB was detected inside the cells (Figs. S3, S17) due to the formation of a nonfluorescent, “closed” lactone isomer, thus minimizing the fluorescence interference from the vehicle to the benefit of multicolour imaging applications. Moreover, PV-1 can not only efficiently deliver 22 different cell-impermeable, organic fluorescent probes into live cells but also simultaneously transfer up to three different probes into live cells for the live-cell imaging of various organelles.” to make the statement more accurate.

**Fig. S17:**
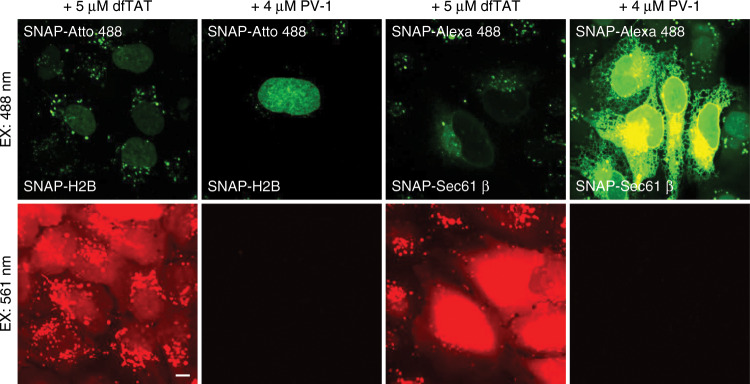
Characterization of the peptide vehicles in live cells. Live U-2 OS cells expressing SNAP-H2B or SNAP-Sec61β were co-incubated with the indicated probes (5 μM) and dfTAT (5 μM) or PV-1 (4 μM) for 1 h before imaging. Imaging acquisition was performed using an Olympus IX83 confocal microscope outfitted with a spinning-disk scan head (PerkinElmer). Scale bars: 50 μm.

**Reference**

36. Brock, D. J. et al. Efficient cell delivery mediated by lipid-specific endosomal escape of supercharged branched peptides. *Traffic* **19**, 421–435 (2018).

We apologize for any inconvenience this may have caused.

