# Supplementary material for: Simple and efficient delivery of cell-impermeable organic fluorescent probes into live cells for live-cell superresolution imaging
Source: Light Sci Appl. 2019 Aug 14;8:73. doi: 10.1038/s41377-019-0188-0 (PMC6804920; doi:10.1038/s41377-019-0188-0)
Supplement: Supplementary file 1 — Supporting Information [file 41377_2019_188_MOESM1_ESM.pdf]

Supplementary Materials for

Simple and efficient delivery of cell-impermeable organic fluorescent probes into live cells for live-cell super-resolution imaging

Meng Zhang[1,2], Meihua Li[1,2], Wenting Zhang[1,2], Yubing Han[1,2], Yu-Hui Zhang[1,2,*]

[1]Britton Chance Center for Biomedical Photonics, Wuhan National Laboratory for Optoelectronics-Huazhong University of Science and Technology, Wuhan, Hubei, 430074, China.

[2]MoE Key Laboratory for Biomedical Photonics, School of Engineering Sciences, Huazhong University of Science and Technology, Wuhan, Hubei, 430074, China.

*Corresponding author. Email: zhangyh@mail.hust.edu.cn

contents

Figure S1 ..... **S2**
Figure S2 ..... **S5**
Figure S3 ..... **S6**
Figure S4 ..... **S7**
Figure S5 ..... **S8**
Figure S6 ..... **S8**
Figure S7 ..... **S9**
Figure S8 ..... **S10**
Figure S9 ..... **S11**
Figure S10 ..... **S12**
Figure S11 ..... **S13**
Figure S12 ..... **S13**
Figure S13 ..... **S14**
Figure S14 ..... **S15**
Figure S15 ..... **S16**
Figure S16 ..... **S16**
Table S1 ..... **S17**
Note S1 ..... **S21**
Note S2 ..... **S23**
Note S3 ..... **S30**
Note S4 ..... **S32**
Note S5 ..... **S33**
Note S6 ..... **S34**
Note S7 ..... **S35**
References **S35**

TP10

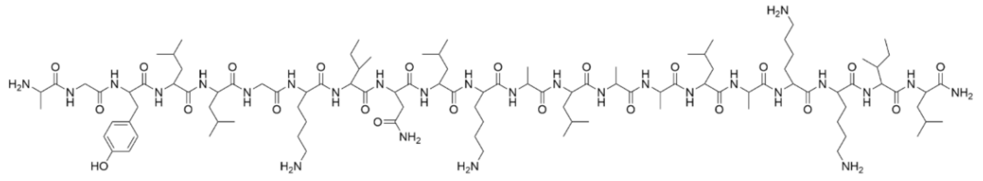

GALA

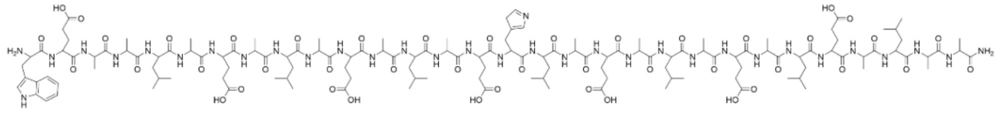

Pene

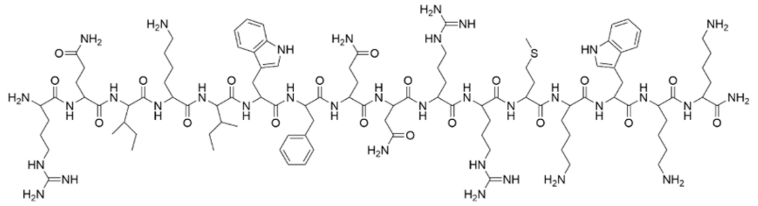

dfTAT

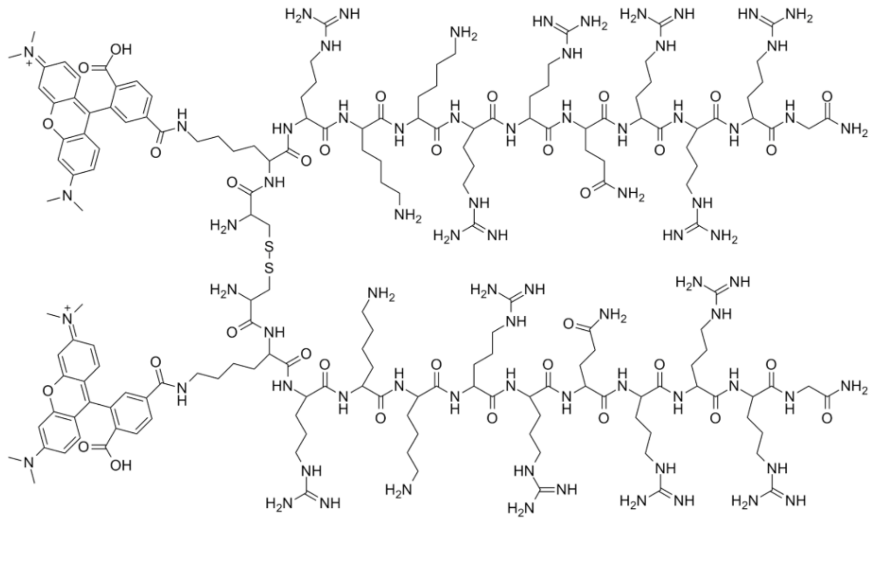

PV-1

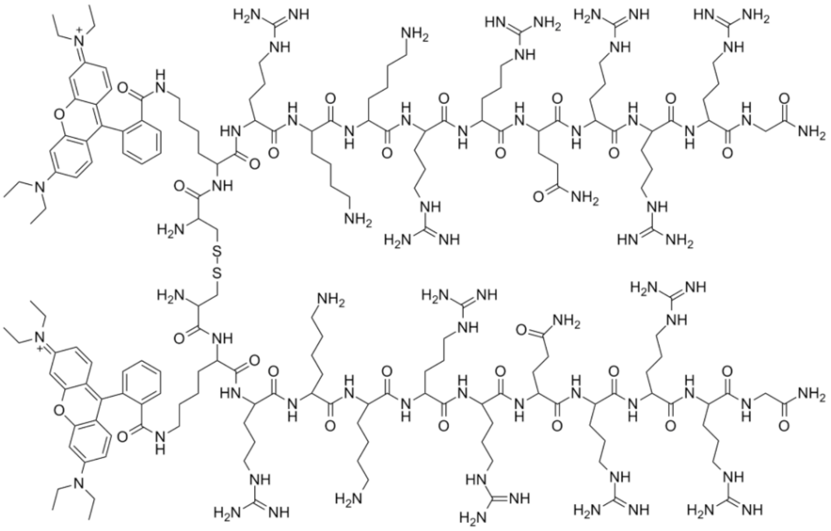

PV-2

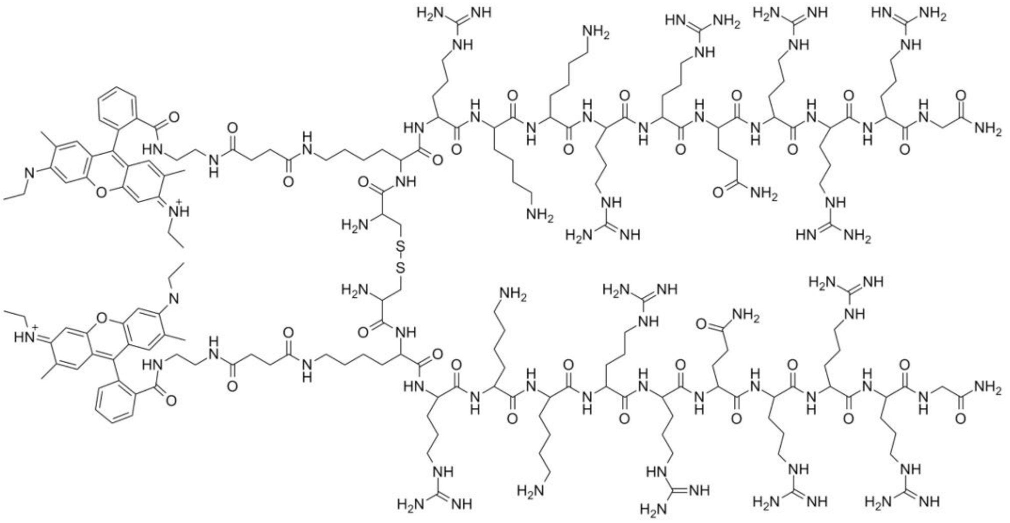

PV-3

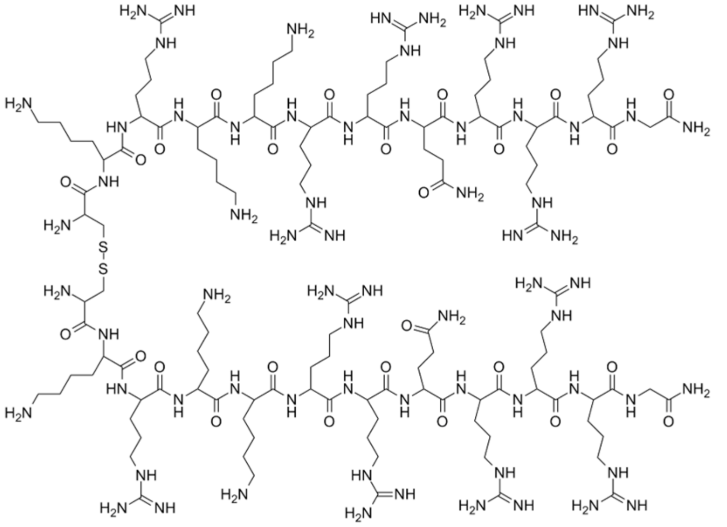

PV-4

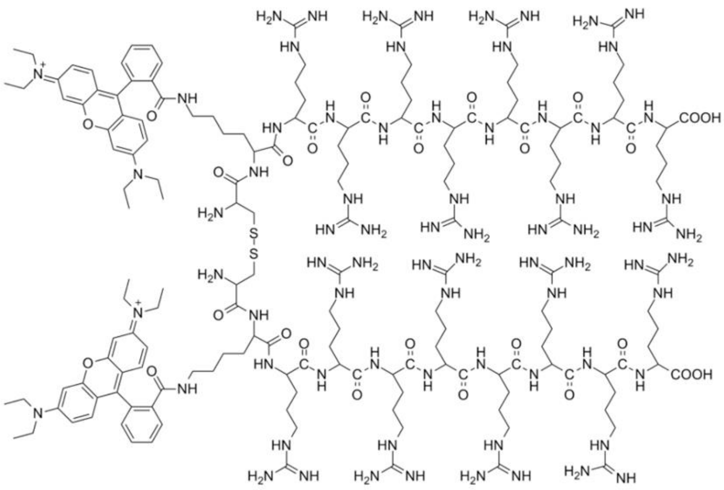

PV-5

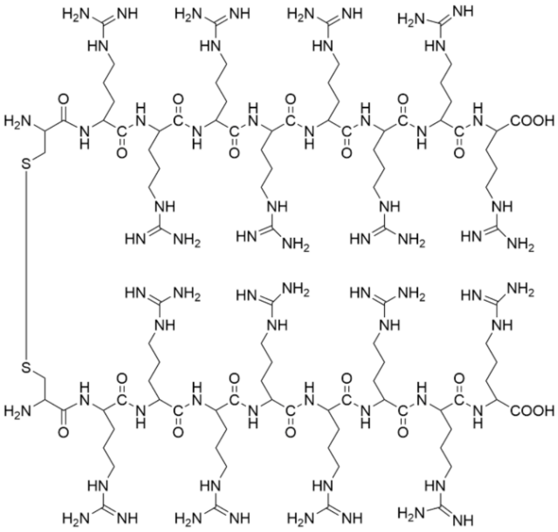

Fig. S1. **The chemical structures of the peptide vehicles.** The synthesis of these peptide vehicles, including TP10, GALA, Pene, dfTAT, PV-1, PV-2, PV-3, PV-4, and PV-5, are shown in Note S1 and S2.

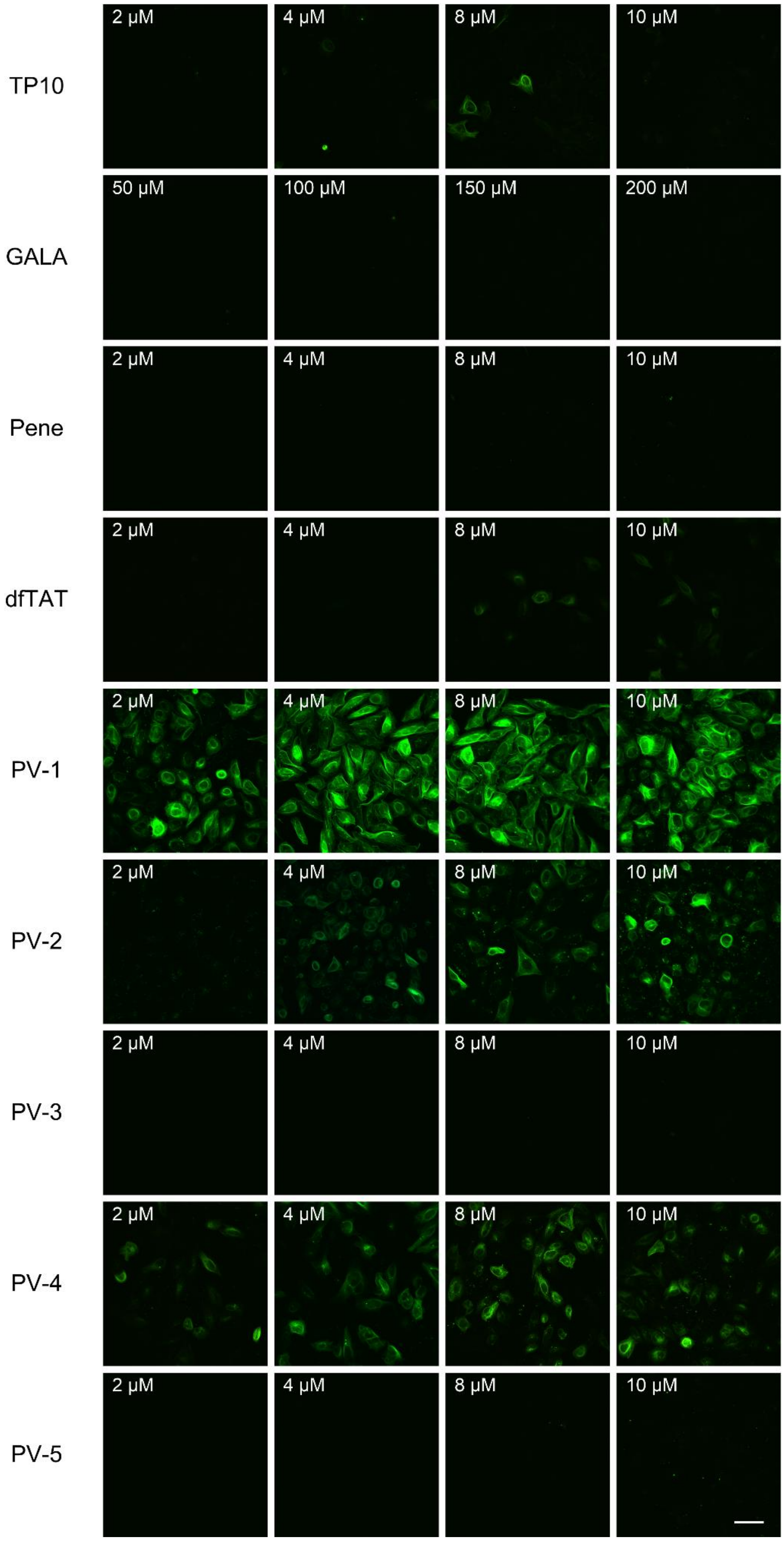

Fig. S2. Characterization of the peptide vehicles at different concentrations in live cells. Confocal microscopy images of live U-2 OS cells after a 1-h co-incubation

with Tubulin-FITC (5 µM) and the indicated peptide vehicles at the indicated concentrations, respectively. Imaging acquisition was performed using an Olympus IX83 confocal microscope outfitted with a spinning-disk scan head (PerkinElmer). Scale bar: 50 µm.

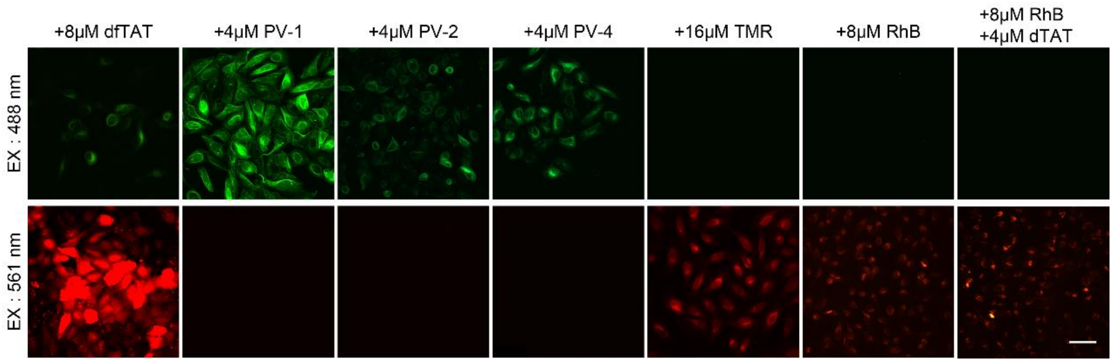

Fig. S3. Characterization of the peptide vehicles excited by a 561 nm laser in live cells. Confocal microscopy images of live U-2 OS cells after a 1-h co-incubation with Tubulin-FITC (5 µM) and the indicated peptide vehicles. Imaging acquisition was performed using an Olympus IX83 confocal microscope outfitted with a spinning-disk scan head (PerkinElmer). Scale bar: 50 µm.

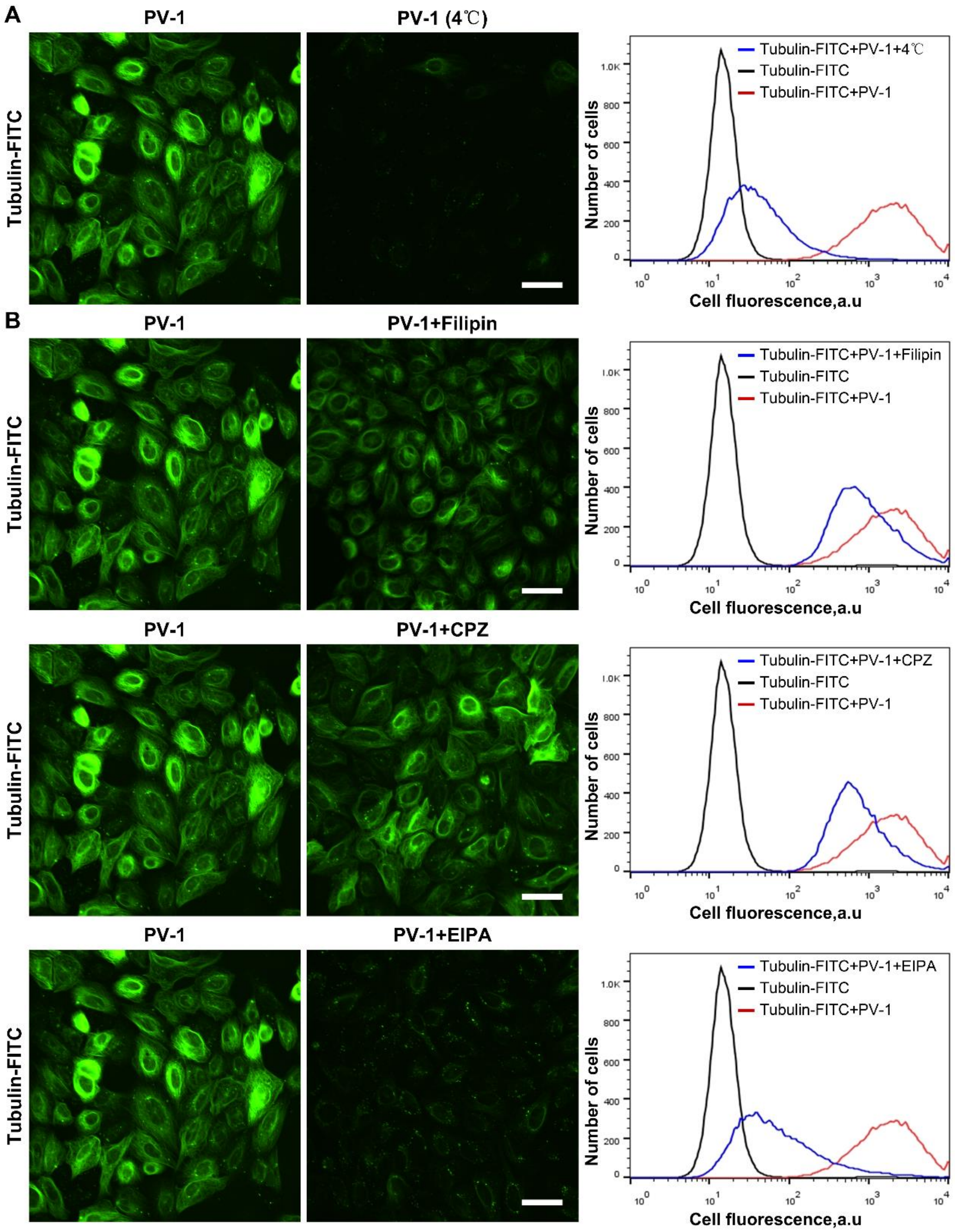

Fig. S4. Studies on the cellular uptake mechanism involved in the delivery of PV-1. (A) Confocal microscopy images and flow cytometry analyses of live U-2 OS cells after co-incubation with Tubulin-FITC (5 μM) and PV-1 (4 μM) for 1 h at 37 °C or 4 ℃. (B) Confocal microscopy images and flow cytometry analyses of live U-2 OS cells after co-incubation with Tubulin-FITC (5 μM) and PV-1 (4 μM) for 1 h at 37 °C in the presence or absence of the indicated inhibitors. Imaging acquisition was performed using an Olympus IX83 confocal microscope outfitted with a spinning-disk scan head (PerkinElmer). Scale bar: 50 μm.

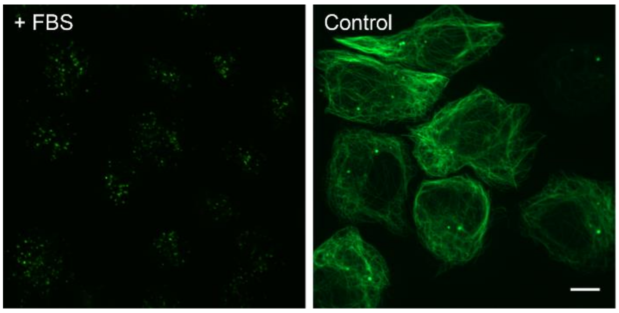

Fig. S5. FBS can affect the delivery ability of PV-1. Confocal microscopy images of live U-2 OS cells after co-incubation with Tubulin-FITC (5 µM) and PV-1 (4 µM) in fresh growth medium with FBS or without FBS. Scale bar: 10 µm.

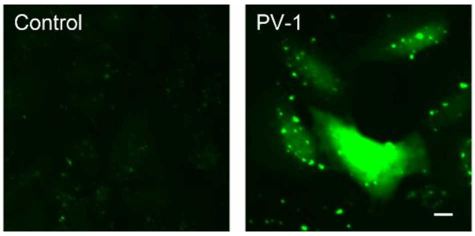

Fig. S6. PV-1 can deliver proteins into living cells. Confocal microscopy images of live U-2 OS cells after a 1-h incubation with EGFP (5 µM) in the absence or presence of PV-1 (4 µM). Scale bar: 10 µm.

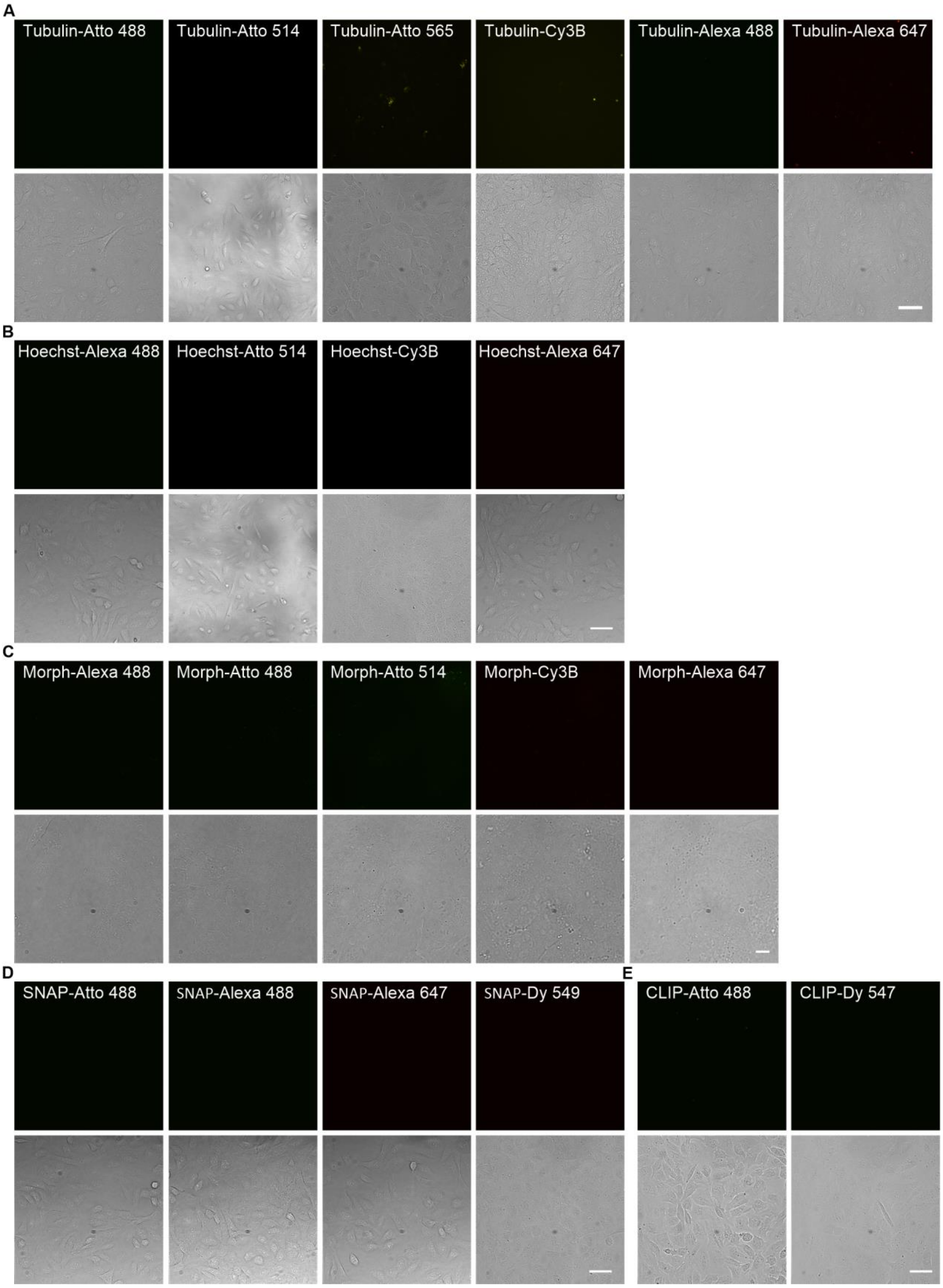

Fig. S7. All of the probes are cell-impermeable without assistance. (A to C) Live U-2 OS cells were incubated with the indicated probes (5 μM) for 1 h before imaging. (D) Live U-2 OS cells expressing SNAP-Sec61β were incubated with the indicated probes (5 μM) for 1 h before imaging. (E) Live U-2 OS cells expressing CLIP-Sec61β were incubated with the indicated probes (5 μM) for 1 h before imaging. The cells stained with Tubulin-Atto 514, Hoechst-Atto 514, or Morph-Atto 514 were imaged by a Zeiss LSM 710 and the others were imaged by an Olympus IX83 confocal

microscope outfitted with a spinning-disk scan head (PerkinElmer). Scale bars: C, 10 μm, A, B, and D: 50 μm.

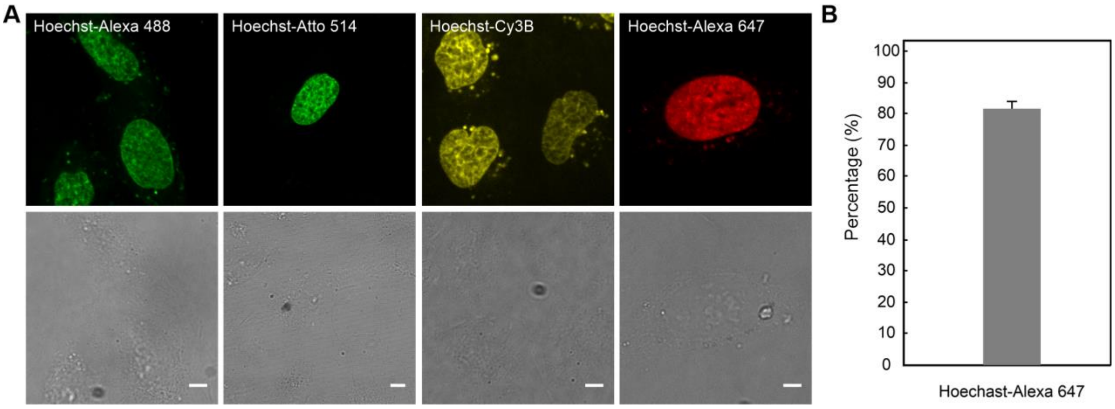

Fig. S8. All the Hoechst probes are efficiently delivered into live cells via co-incubation with PV-1 and specifically labeled nuclei. (A) Confocal microscopy images of live U-2 OS cells after a 1-h co-incubation with PV-1 (4 μM) and the indicated probe (3 μM). Scale bars: 50 μm. (B) The percentage of live U-2 OS cells labeled by 3 μM Hoechst-Alexa 647 after a 1-h co-incubation with PV-1 (4 μM) (n = 1,500 cells for each experiment). The error bars represent the standard deviations of triplicate experiments.

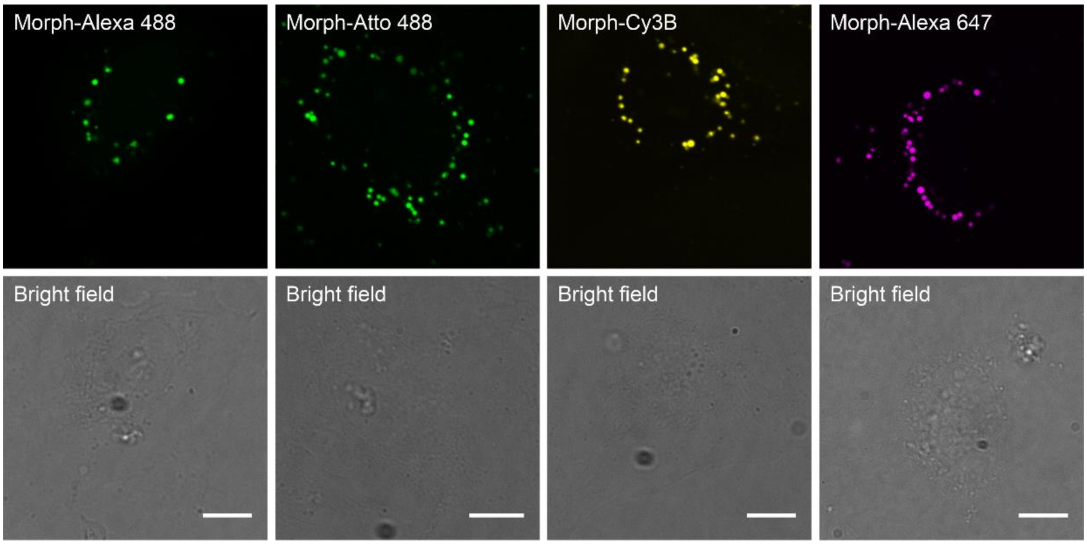

Fig. S9. The Morph probes are efficiently delivered into live cells via co-incubation with PV-1 and specifically labeled lysosomes. Live U-2 OS cells were co-incubated with the indicated probe (5 μM) and PV-1 (4 uM) for 1 h. The cells were imaged by an Olympus IX83 confocal microscope outfitted with a spinning-disk scan head (PerkinElmer). Scale bars: 10 μm.

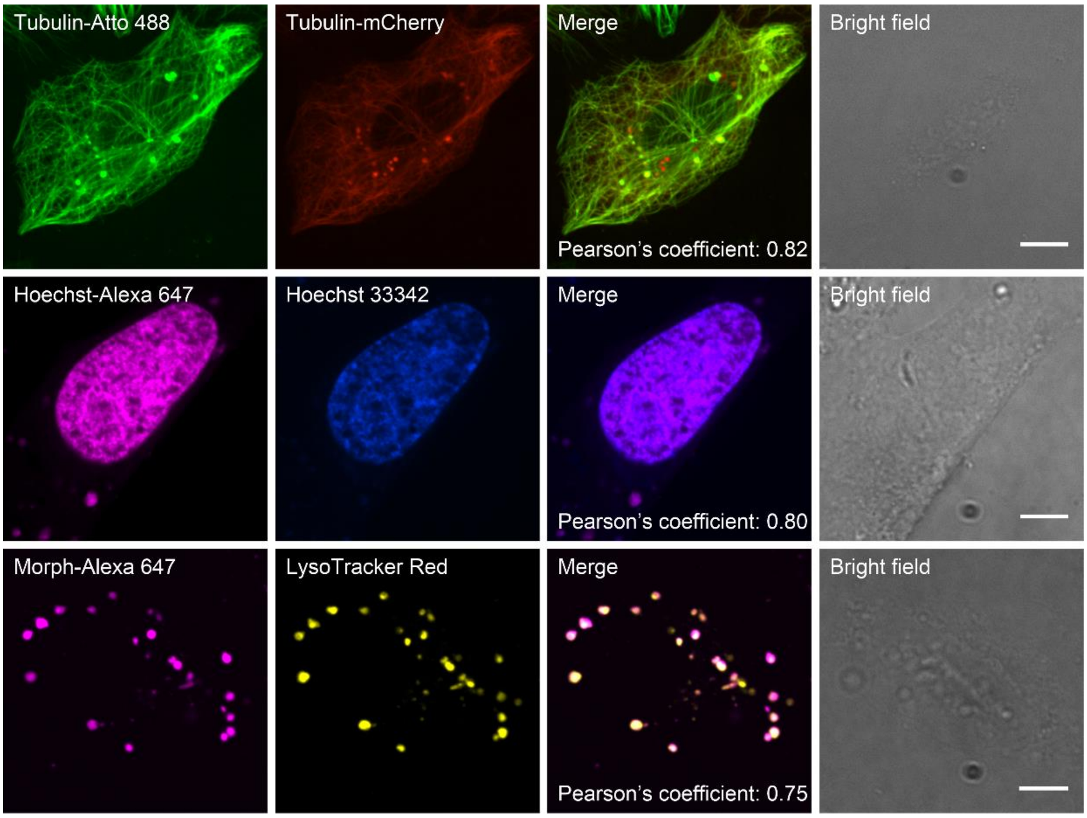

Fig. S10. Co-localization studies by using Tubulin-mCherry, Hoechst33342, and LysoTracker Red as the standard markers for microtubules, nuclei, and lysosomes, respectively. Scale bars: 10 μm.

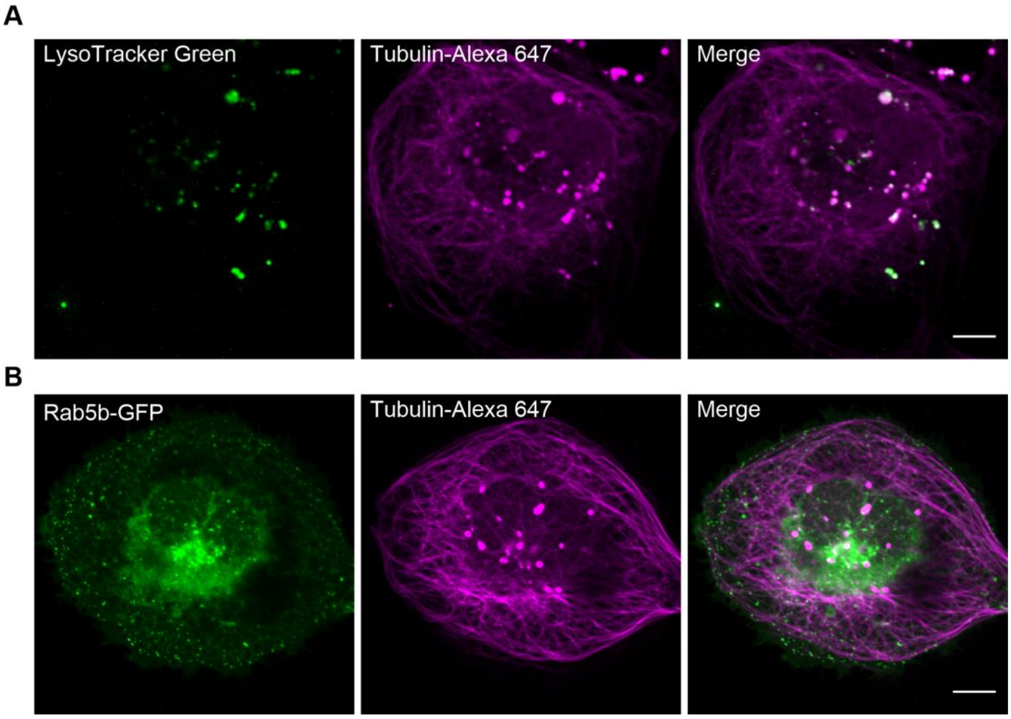

Fig. S11. Co-localization studies of Tubulin-Alexa 647 with LysoTracker Green and Rab5b-GFP. (A) Live U-2 OS cells were co-incubated with Tubulin-Alexa 647 (5 uM) and PV-1 (4 uM) for 1 h. Then lysosomes were labeled with LysoTracker Green (500 nM) for 30 min before imaging. (B) Live U-2 OS cells transiently transfected by Rab5b-GFP were co-incubated with Tubulin-Alexa 647 (5 μM) and PV-1 (4 uM) for 1 h before imaging. Imaging acquisition was performed using an Olympus IX83 confocal microscope outfitted with a spinning-disk scan head (PerkinElmer). Scale bars: 10 μm.

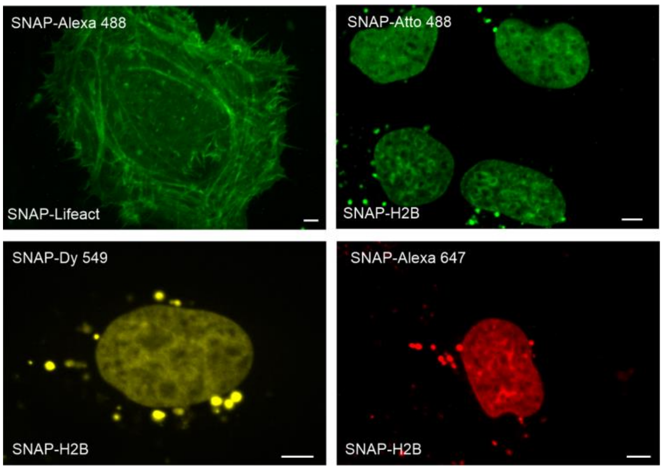

Fig. S12. SNAP probes are delivered into live cells via co-incubation with PV-1 and specifically labeled F-actin or nuclei. Live U-2 OS cells expressing SNAP-Lifeact or SNAP-H2B were co-incubated with PV-1 (4 μM) and the indicated probes (5 μM) for 1 h before imaging. The expression plasmids are indicated. Imaging acquisition was performed using an Olympus IX83 confocal microscope outfitted with a spinning-disk scan head (PerkinElmer). Scale bars: 5 μm.

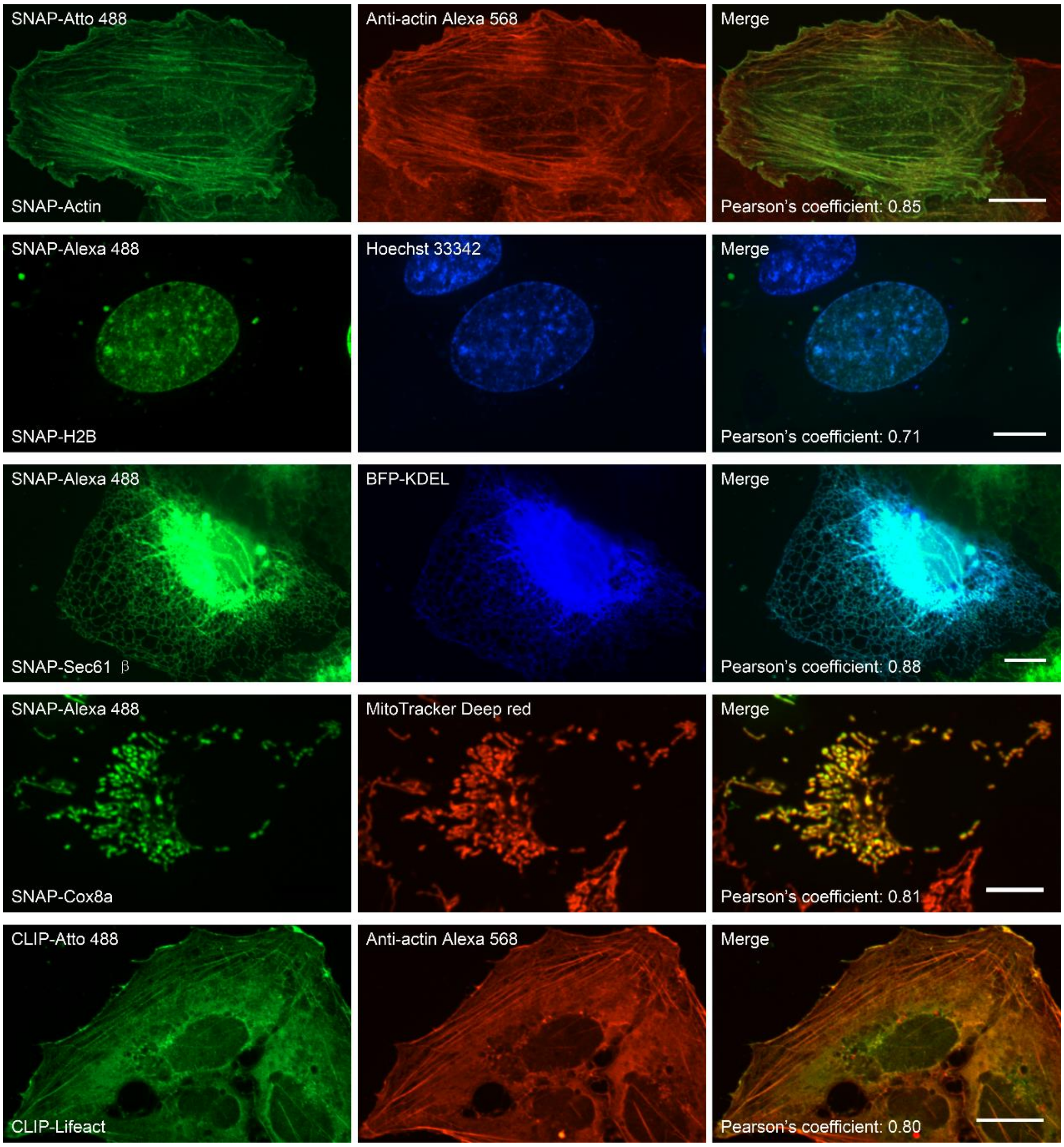

Fig. S13. Co-localization studies by using anti-actin antibody Alexa 568, Hoechst33342, BFP-KDEL, and MitoTracker Deep Red as the standard markers for F-actin, nuclei, the ER, and mitochondria, respectively. Scale bars: 10 μm.

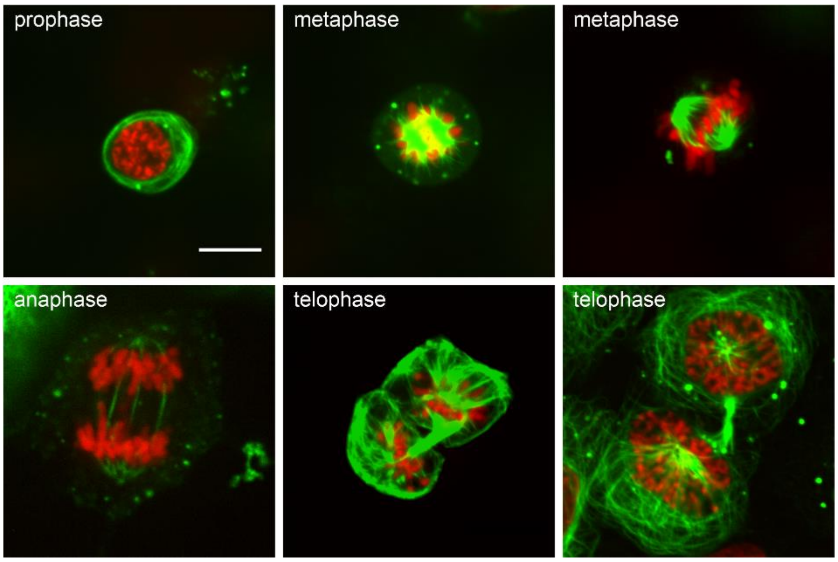

Fig. S14. Live cells at different stages of the cell cycle are labeled. Confocal microscopy images of live U-2 OS cells after a 1-h co-incubation with PV-1 (4 µM), Tubulin-Atto 488 (5 µM), and Hoechst-Alexa 647 (3 µM). Scale bar: 10 µm.

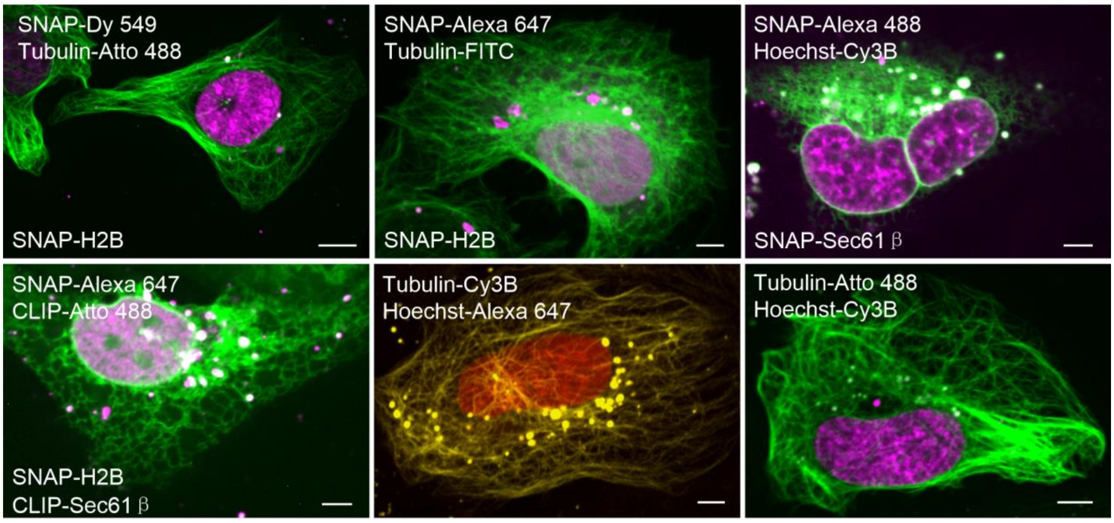

Fig. S15. PV-1 simultaneously delivers two cell-impermeable organic fluorescent probes into live cells. Live U-2 OS cells expressing SNAP-H2B, SNAP-Sec61β, or/and CLIP-Sec61β (top row and bottom right) or wide type (bottom, last two images) were co-incubated with PV-1 (4 μM) and the two indicated probes (Hoechst-Alexa 647 and Hoechst-Alexa Cy3B: 3μM, the others: 5 μM). The expression plasmids are indicated. Scale bars: 5 μm.

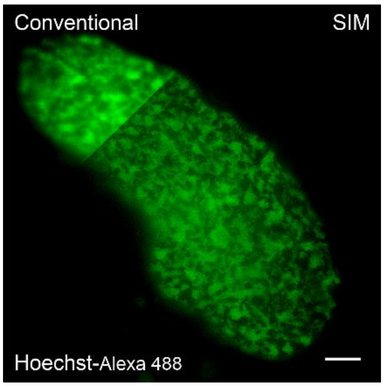

Fig. S16. A live-cell SIM image of nucleus. Diffraction-limited (upper left) and SIM (lower right) image of live U-2 OS cells after a 1-h co-incubation with PV-1 (4 μM) and Hoechst-Alexa 488 (3 μM). Scale bar: 2 μm.

Table S1. The chemical structures and optical properties of the probes used in this research.

Target	Name	Structure	Ex (nm)	Em (nm)	Synthesis
microtubule	Tubulin-FITC	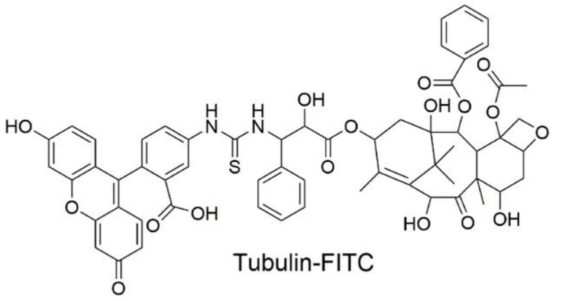	488	502	Note S3 and S4
	Tubulin-Atto 488	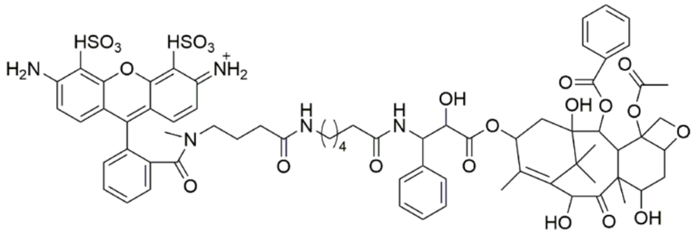	498	520	
	Tubulin-Alexa 488	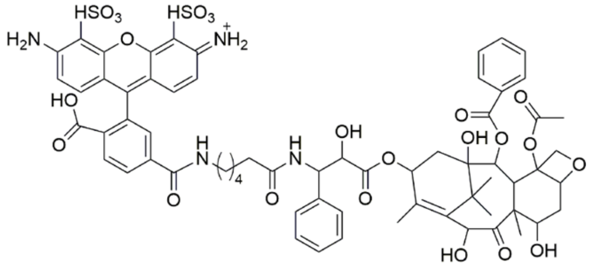	490	525	
	Tubulin-Atto 514	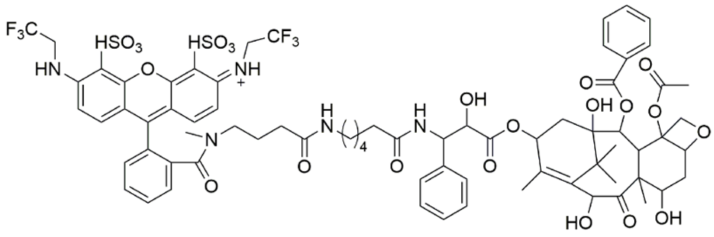	511	533	
	Tubulin-Atto 565	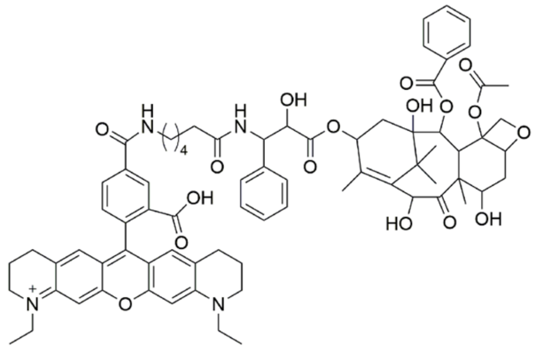	565	590	

	Tubulin-Cy3B	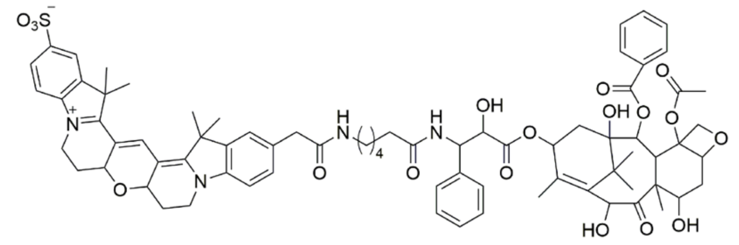	559	570	
	Tubulin-Alexa 647	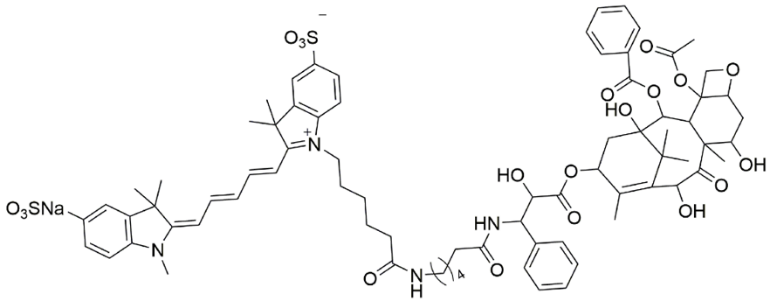	651	672	
nuclei	Hoechst-Alexa 488	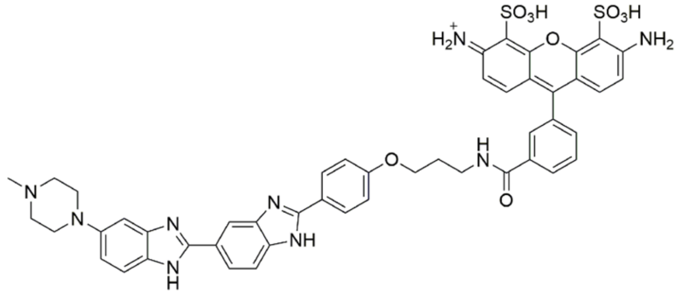	490	525	Note **S5** and **S6**
	Hoechst-Atto 514	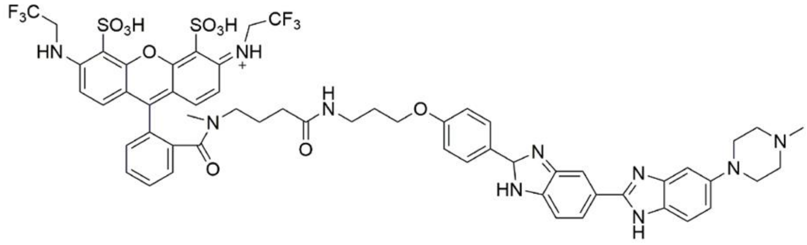	511	533	
	Hoechst-Cy3B	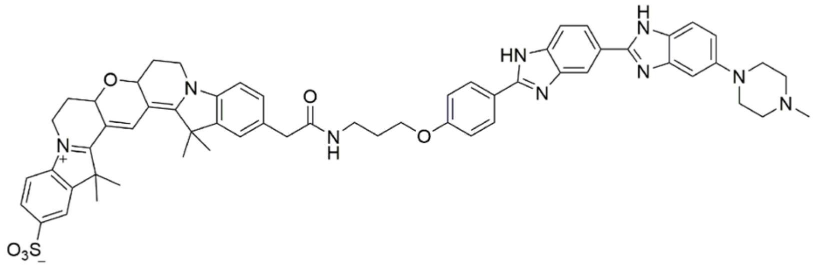	559	570	
	Hoechst-Alexa 647	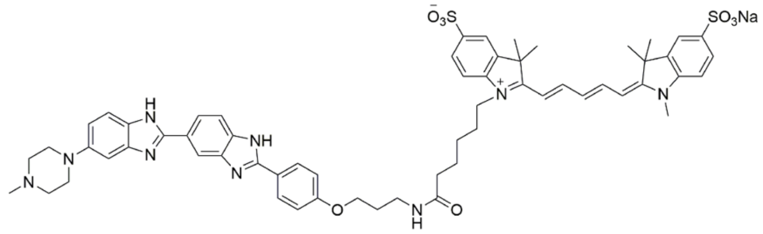	651	672	

lysosomes	Morph-Atto 488	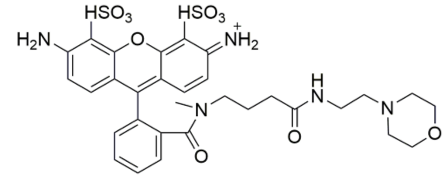	498	520	Note S7
	Morph-Alexa 488	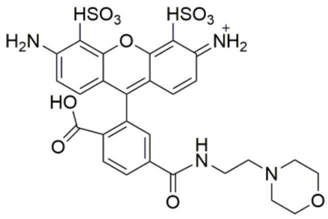	490	525	
	Morph-Atto 514	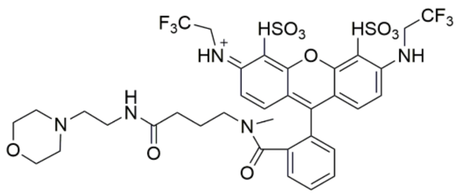	511	533	
	Morph-Cy3B	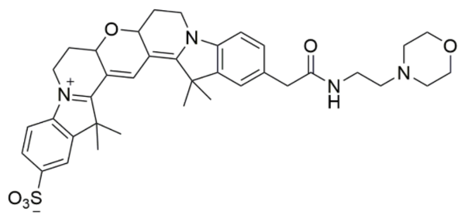	559	570	
	Morph-Alexa 647	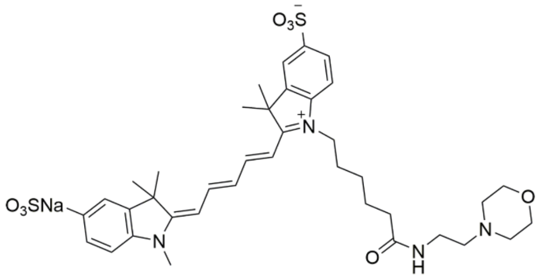	651	672	
SNAP-tag®	SNAP-Atto 488	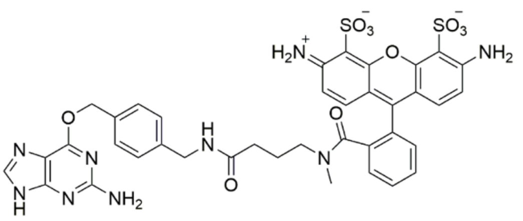	506	526	commercial available New England Biolabs inc.
	SNAP-Alexa 488	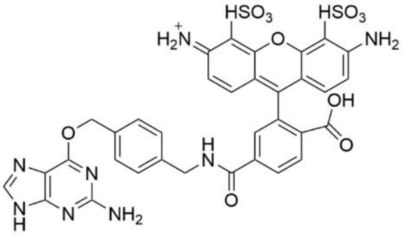	496	520	

	SNAP-<br>Dy 549	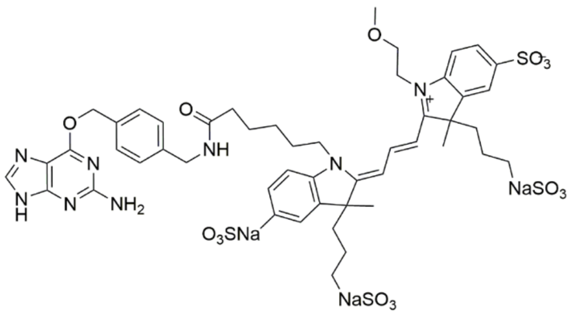	560	575	
	SNAP-<br>Alexa 647	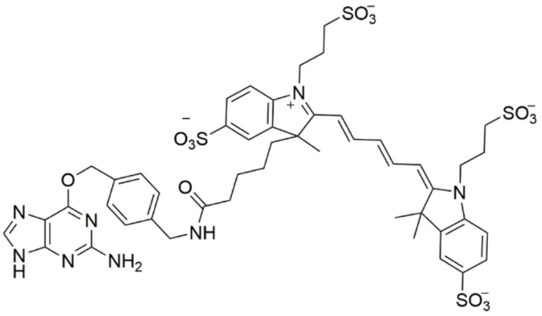	652	670	
**CLIP-tag®**	CLIP-<br>Atto 488	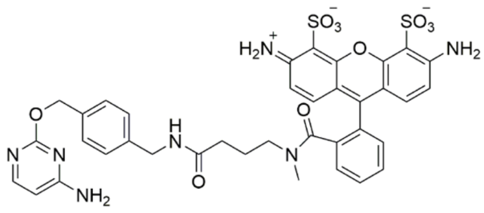	506	526	**Commercial available<br>New England Biolabs inc.**
	CLIP-<br>Dy 547	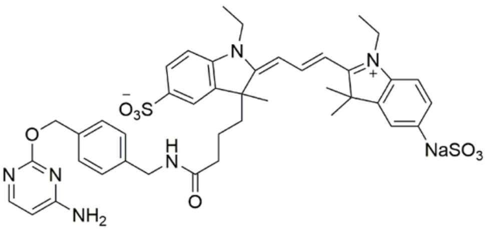	554	568	

Note S1. The synthesis procedures of the peptide vehicles.

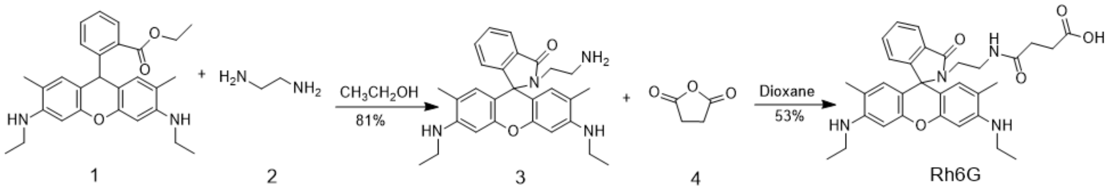

Preparation of 3. To a stirred solution of compound **1** (935 mg, 2 mmol) in ethanol (20 mL), **2** (0.67 mL, 10 mmol) was added. The mixture was refluxed for 6 h until the fluorescence of the solution was disappeared. Then the mixture was cooled to room temperature and the precipitate was collected and washed with cold ethanol. The crude product was purified by recrystallization from acetonitrile to give **3** as a white solid (749 mg, 81% yield). ^{1}H NMR (400 MHz, $CDCl_3$) δ 7.92 (d, 1 H, J = 8 Hz), 7.45 (t, 2 H, J = 4 Hz), 7.05 (d, 1 H, J = 4 Hz), 6.34 (s, 2 H), 6.22 (s, 2 H), 3.52 (s, 2 H), 3.19 (m, 6 H), 2.35 (t, 2 H, J = 6 Hz), 1.90 (s, 6 H), 1.32 (t, 6 H, J = 8 Hz), 1.05 (s, 2 H).

Preparation of Rh6G. To a stirred solution of **4** (212 mg, 2 mmol) in dioxane (10 mL), **3** (914 mg, 2 mmol) in dioxane (10 mL) was slowly added. The solution was warmed to 80 ℃ and kept stirring for 30 min. Then the reaction was cooled to room temperature. The white crystal was filtered and dried under reduced pressure. The residue was purified by recrystallized from dioxane to give Rh6G (590 mg, 53% yield). ^{1}H NMR (400 MHz, $CDCl_3$) δ 7.92 (t, 1 H, J = 4 Hz), 7.48 (t, 2 H, J = 4 Hz), 7.06 (t, 2 H, J= 4 Hz), 6.35(s, 2 H), 6.20 (s, 2 H), 3.28 (d, 2 H, J = 8 Hz), 3.20 (dd, 2 H, J = 8 Hz, 2 Hz), 2.98 (d, 2 H, J = 8 Hz), 2.59 (d, 2 H, J = 8 Hz), 2.43 (d, 2 H, J = 8 Hz), 1.90 (s, 6 H), 1.32 (t, 6H, J = 8 Hz).

Preparation of Tp10, GALA, Pene, dfTAT, PV-1, PV-2, PV-3, PV-4, PV-5. Tp10, GALA, Pene, dfTAT, were synthesized as previously reported[17-20] using corresponding dyes. The HPLC analysis and ESI mass spectrum are shown in **Note S2**.

PV-1, PV-2, PV-3, PV-4, and PV-5 were generated by the dimerization of the corresponding precursors. PV-1 precursor, PV-2 precursor and PV-3 precursor were synthesized using standard Fmoc protocols by solid-phase peptide synthesis (SPPS) on rink amide MBHA resin. Fmoc-Lys(Boc)-OH, Fmoc-Arg(Pbf)-OH, Fmoc-Lys(Mtt)-OH, Fmoc-Gly-OH, Fmoc-Gln(Trt)-OH and Fmoc-Cys(Trt)-OH were used to synthesis the peptide. Reactions were carried out in a SPPS vessel at room temperature using a stream of dry N_2 to provide agitation. The Fmoc protecting group was cleaved with 20% piperidine in DMF added to the Fmoc-peptide resin (1 eq). Deprotection reactions were carried out once for 5 min and 15 min with a washing step in between reactions. Amino acid coupling reactions were carried out for 4 h with a mixture of Fmoc-amino acid (4 eq), HBTU (3.7eq) and di-isopropylethylamine (DIEA) (10 eq) in dimethylformamide (DMF) added to the resin. Upon completion of the reactions, the resin was washed with DMF and dichloromethane (DCM) .The Mtt protecting group was cleaved with 2% trifluoroacetic acid (TFA) and 2%

tri-isopropylsilane (TIS) in DCM, and the resin was washed with DCM and DMF. To couple PV-1 precursor with RhB or to couple PV-2 precursor with Rh6G derivative, a mixture of RHB or Rh6G derivative (4 eq), HBTU (3.9 eq) and DIEA (10 eq) in DMF was added to the resin, the reaction was stirred overnight. Following peptide assembly, the resin was washed with DCM and dried in vacuo. To achieve global deprotection and cleavage from the resin, the resin was treated with 2.5 % ethanedithiol (EDT), 2.5 % TIS and 2.5 % H_2O in TFA for 3 h. The crude peptide was precipitated and washed with cold anhydrous ether. The precipitate was purified by reverse-phase HPLC (solvent A: 0.1% TFA in acetonitrile, solvent B: 0.1% TFA in water). Then the purified peptide (PV-1 precursor, PV-2 precursor or PV-3 precursor) and O_2 were dissolved in phosphate buffer saline (PBS), pH 7.4. The reaction was stirred overnight. The products were purified by reverse-phase HPLC (solvent A: 0.1% TFA in acetonitrile, solvent B: 0.1% TFA in water). The HPLC analysis and ESI mass spectrum are shown in **Note S2**.

PV-4 precursor and PV-5 precursor were synthesized using standard Fmoc protocols by solid-phase peptide synthesis (SPPS) on 2-chlorotritylchloride resin. Fmoc-D-Arg(Pbf)-OH,Fmoc-L-Arg(Pbf)-OH, Fmoc-Lys(Dde)-OH and Fmoc-Cys(Trt)-OH were used to synthesis the peptides. Reactions were carried out at room temperature under the atmosphere of N2. Amino acid coupling reactions were carried out for 1.5 h with a mixture of Fmoc-amino acid (3eq), HOBT (3eq), HBTU (3eq) and di-isopropylethylamine (DIEA) (6eq) in DMF. The Fmoc protecting group was cleaved with 20 % piperidine in DMF to the Fmoc-peptide resin (1eq). Deprotection reactions were carried out once for 5 min and 15 min with a washing step in between reactions. To label PV-4 precursor with RhB, the Dde protecting group was cleaved with 2% hydrazinium hydroxide in DMF, and the resin was washed with DCM and DMF. A mixture of RhB (3eq), HOBT (3eq), HBTU (3eq) and DIEA (6eq) in DMF was added to the resin, the reaction was stirred overnight. Following peptide assembly, the resin was washed with DCM and dried in vacuo. To achieve global deprotection and cleavage from the resin, the resin was treated with 2% TIS in TFA for 3 h. The crude peptide was precipitated in cold anhydrous Ether and lyophilized. Then the crude peptide was purified by reverse-phase HPLC (solvent A: 0.1% TFA in acetonitrile, solvent B: 0.1% TFA in water). The purified peptide (PV-4 precursor or PV-5) and O2 were dissolved in phosphate buffer saline (PBS), pH 7.4. The reaction was stirred overnight. The products were purified by reverse-phase HPLC (solvent A: 0.1% TFA in acetonitrile, solvent B: 0.1% TFA in water) to reach a purity > 95%. The HPLC analysis and ESI mass spectrum are shown in **Note S2**.

Note S2. Characterization of Tp10, GALA, Pene, dfTAT, PV-1, PV-2, PV-3, PV-4, and PV-5.

(**a**)

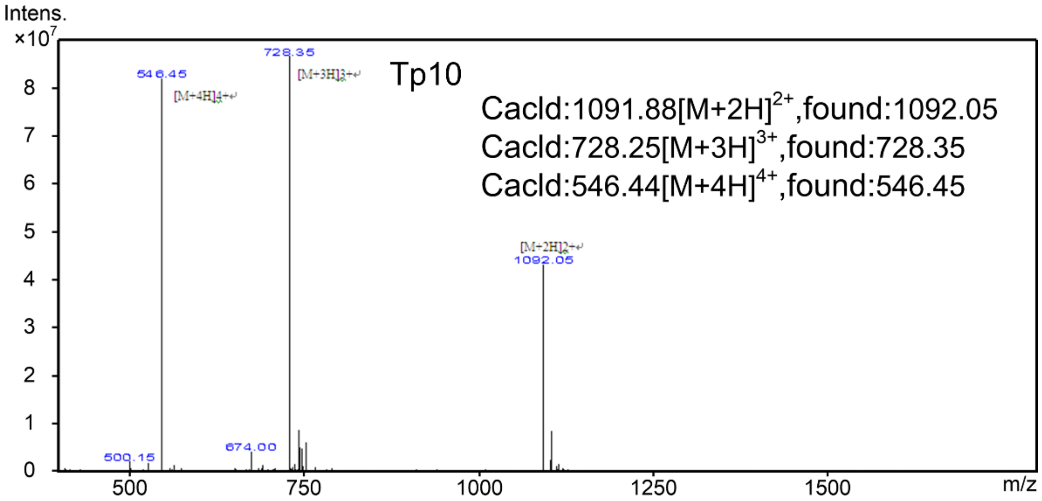

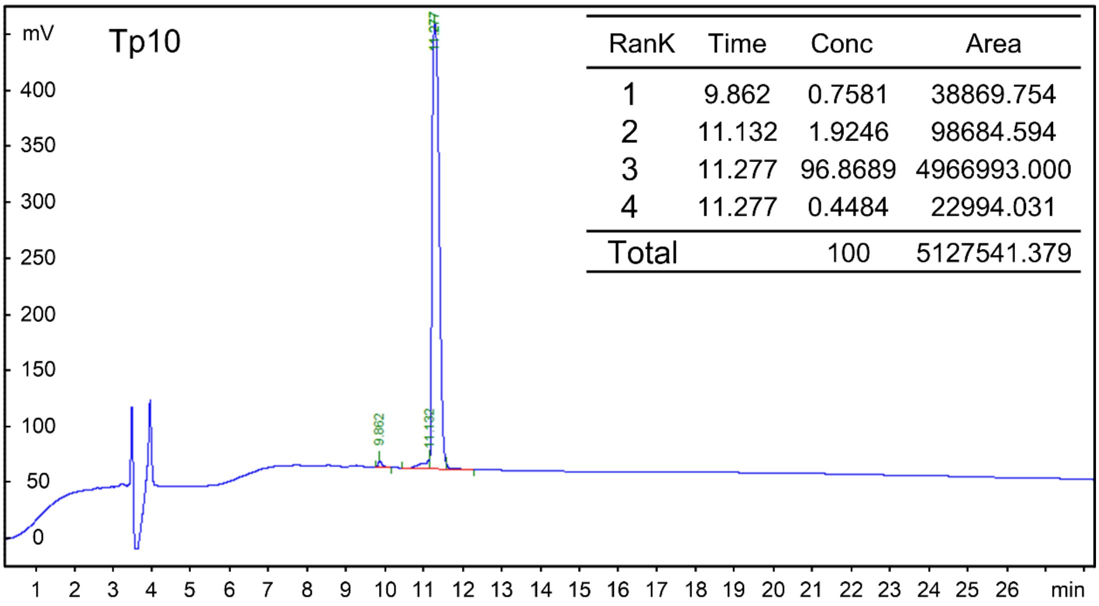

RanK	Time	Conc	Area
1	9.862	0.7581	38869.754
2	11.132	1.9246	98684.594
3	11.277	96.8689	4966993.000
4	11.277	0.4484	22994.031
Total		100	5127541.379

(**b**)

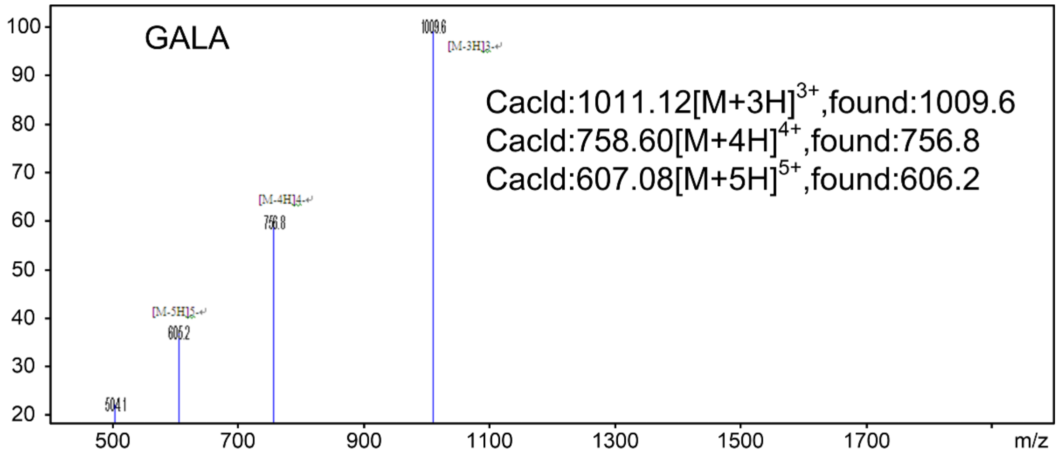

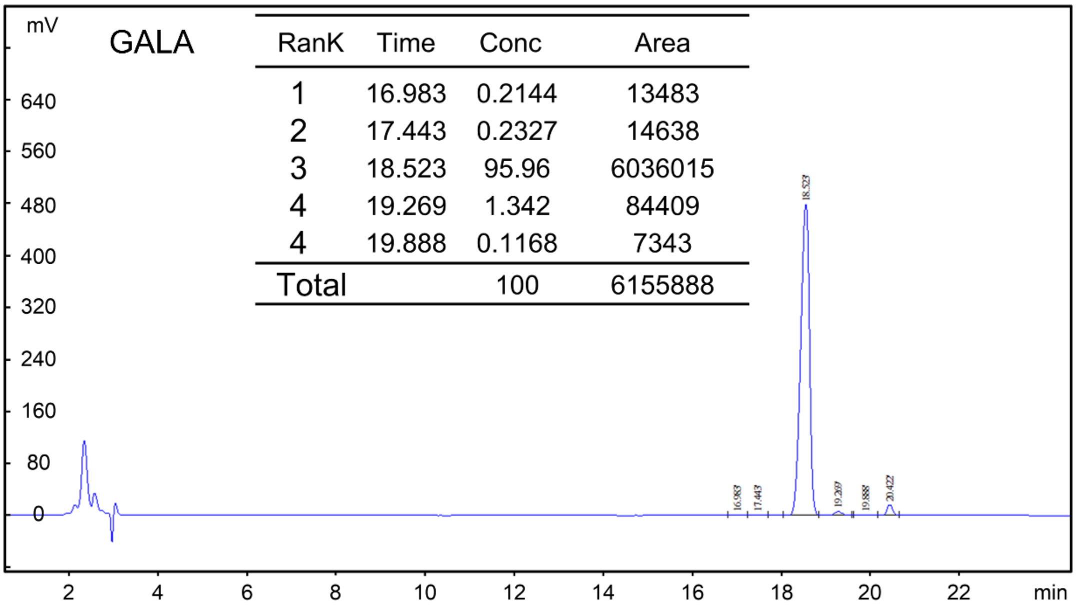

RanK	Time	Conc	Area
1	16.983	0.2144	13483
2	17.443	0.2327	14638
3	18.523	95.96	6036015
4	19.269	1.342	84409
4	19.888	0.1168	7343
Total		100	6155888

(c)

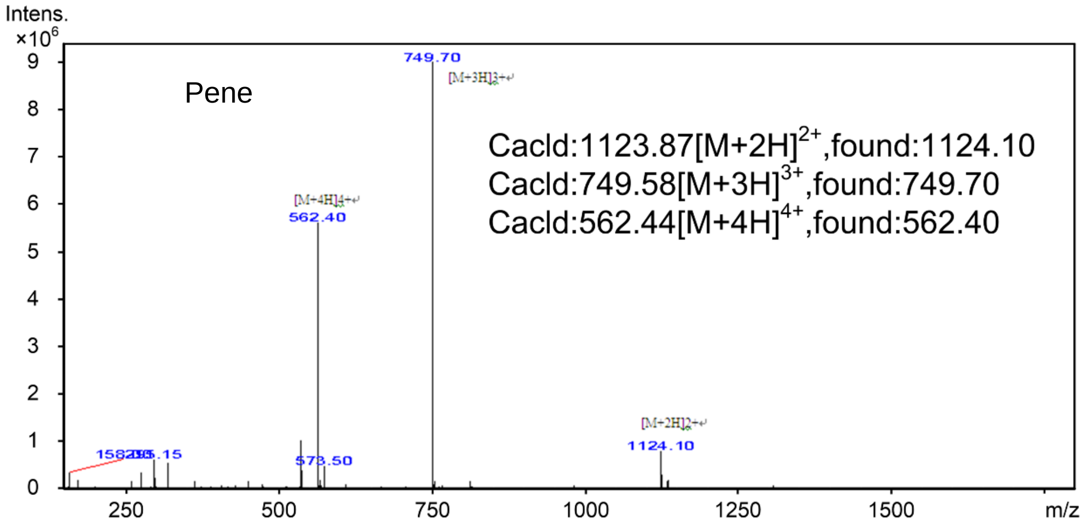

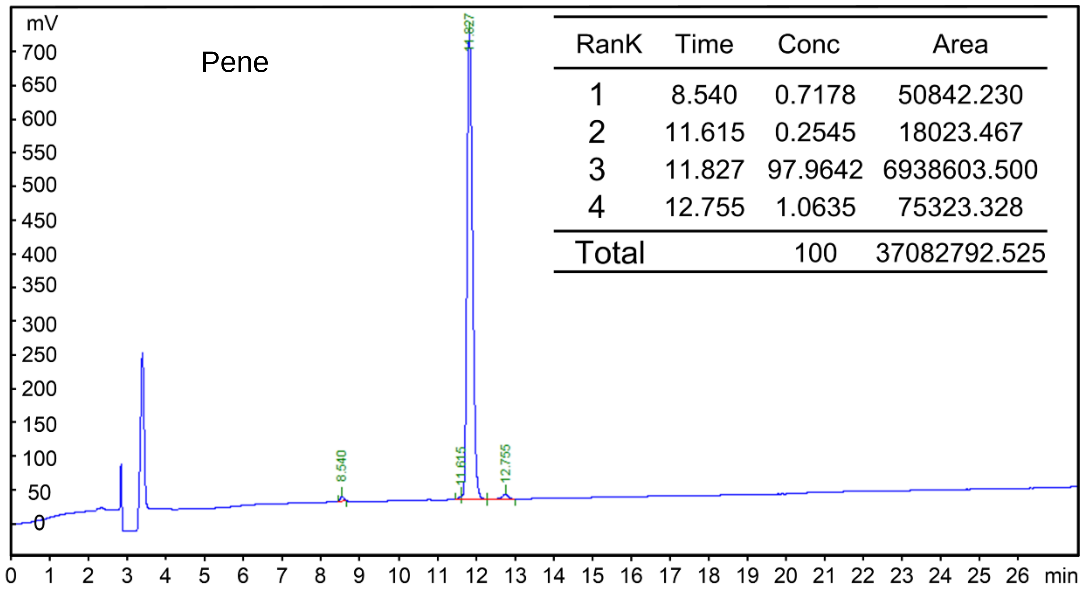

RanK	Time	Conc	Area
1	8.540	0.7178	50842.230
2	11.615	0.2545	18023.467
3	11.827	97.9642	6938603.500
4	12.755	1.0635	75323.328
Total		100	37082792.525

(d)

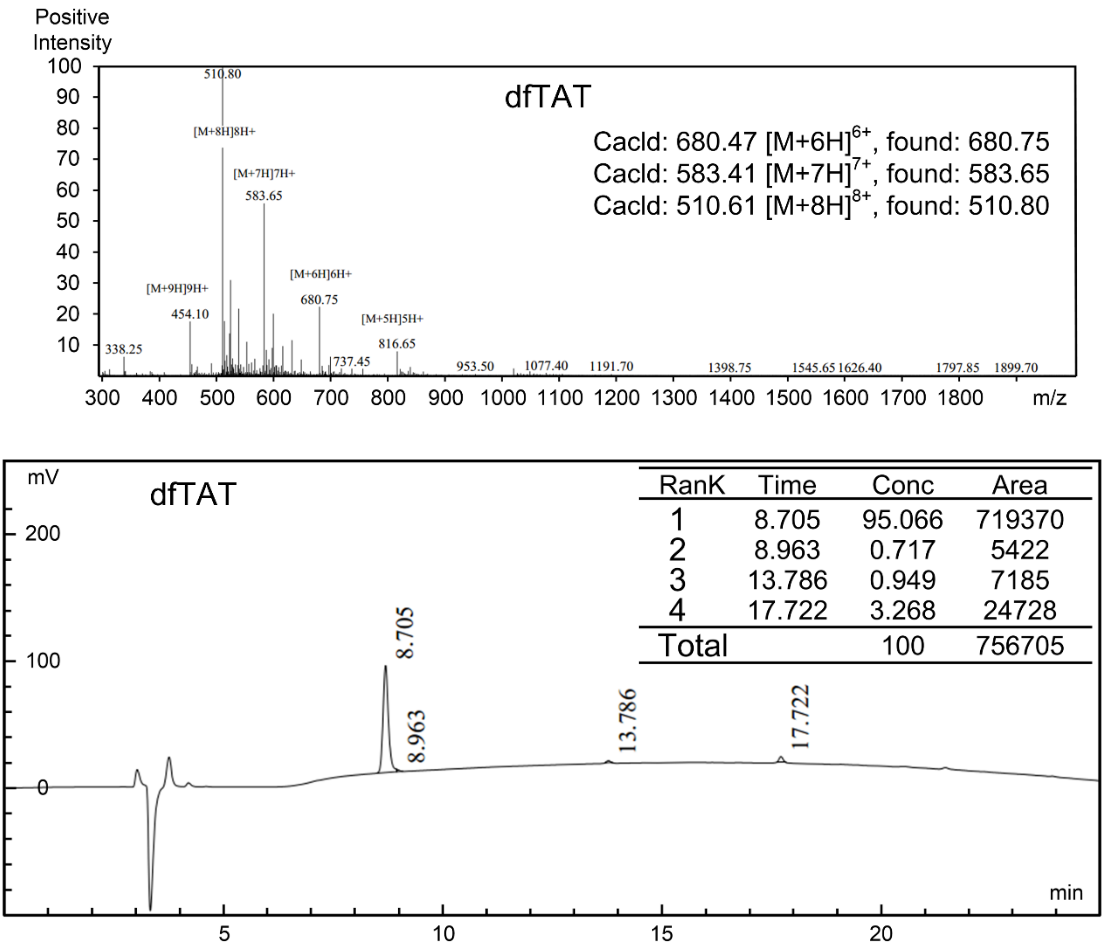

Positive Intensity
dfTAT
Cacld: 680.47 [M+6H]6+, found: 680.75
Cacld: 583.41 [M+7H]7+, found: 583.65
Cacld: 510.61 [M+8H]8+, found: 510.80
[M+8H]8H+
510.80
[M+7H]7H+
583.65
[M+6H]6H+
680.75
[M+9H]9H+
454.10
[M+5H]5H+
816.65
338.25
737.45
953.50
1077.40
1191.70
1398.75
1545.65 1626.40
1797.85
1899.70
m/z
mV
dfTAT
RanK Time Conc Area
1 8.705 95.066 719370
2 8.963 0.717 5422
3 13.786 0.949 7185
4 17.722 3.268 24728
Total 100 756705
8.705
8.963
13.786
17.722
min

(e)

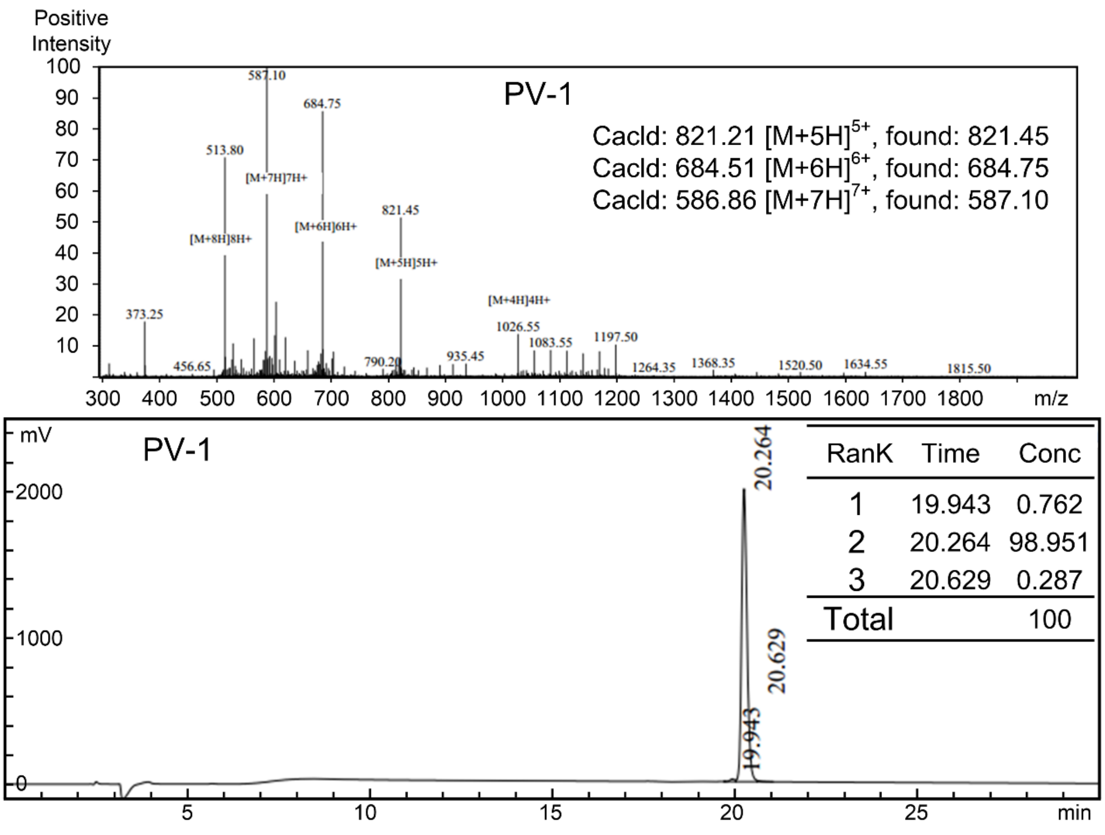

Positive Intensity
PV-1
Cacld: 821.21 [M+5H]5+, found: 821.45
Cacld: 684.51 [M+6H]6+, found: 684.75
Cacld: 586.86 [M+7H]7+, found: 587.10
587.10
684.75
513.80
[M+7H]7H+
821.45
[M+6H]6H+
[M+8H]8H+
[M+5H]5H+
[M+4H]4H+
373.25
1026.55
1083.55
1197.50
456.65
790.20
935.45
1264.35
1368.35
1520.50
1634.55
1815.50
m/z
mV
PV-1
RanK Time Conc
1 19.943 0.762
2 20.264 98.951
3 20.629 0.287
Total 100
20.264
20.629
19.943
min

(f)

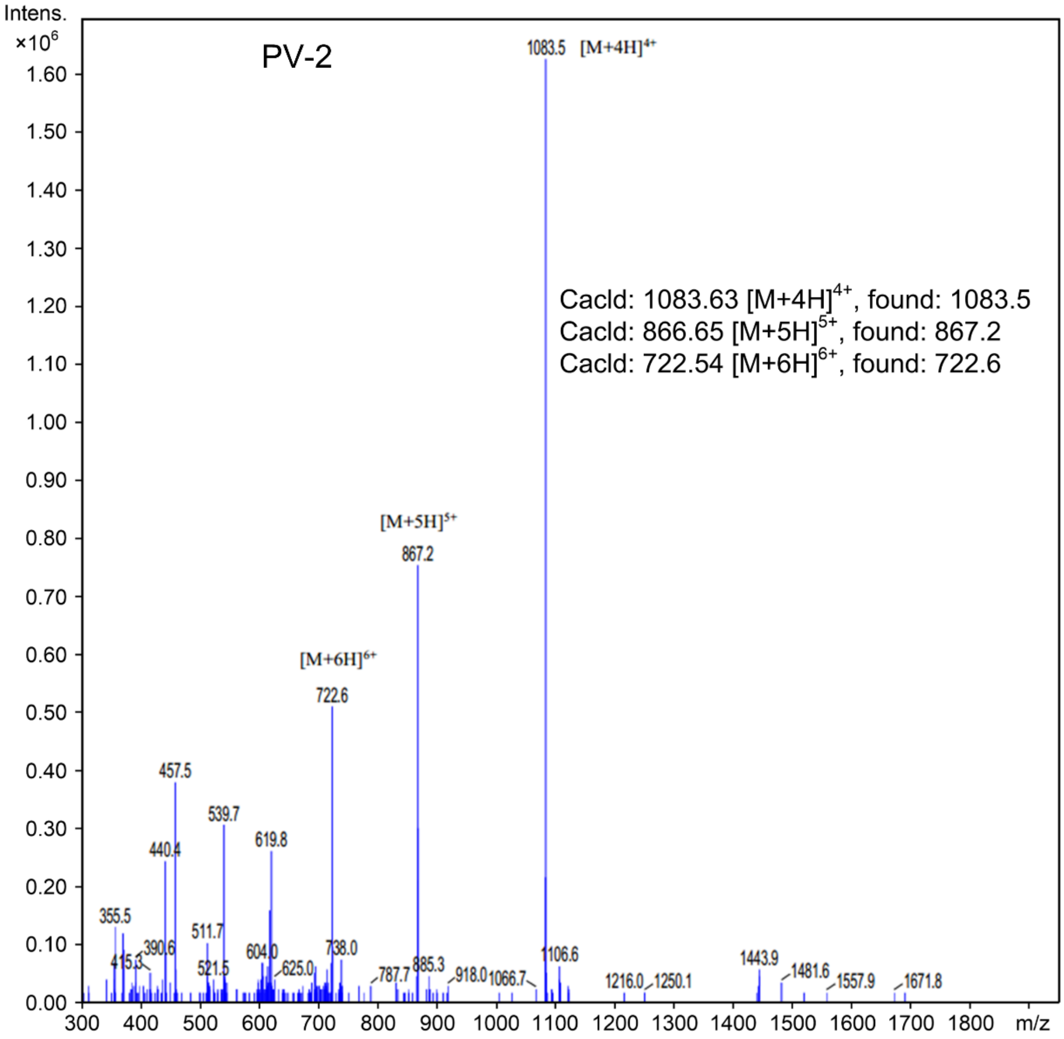
PV-2
Intens.
×10^6
1083.5 [M+4H]4+
Cacld: 1083.63 [M+4H]4+, found: 1083.5
Cacld: 866.65 [M+5H]5+, found: 867.2
Cacld: 722.54 [M+6H]6+, found: 722.6
[M+5H]5+
867.2
[M+6H]6+
722.6
457.5
539.7
619.8
440.4
355.5
511.7
390.6
415.3
521.5
604.0
625.0
738.0
787.7
885.3
918.0
1066.7
1106.6
1216.0
1250.1
1443.9
1481.6
1557.9
1671.8
m/z

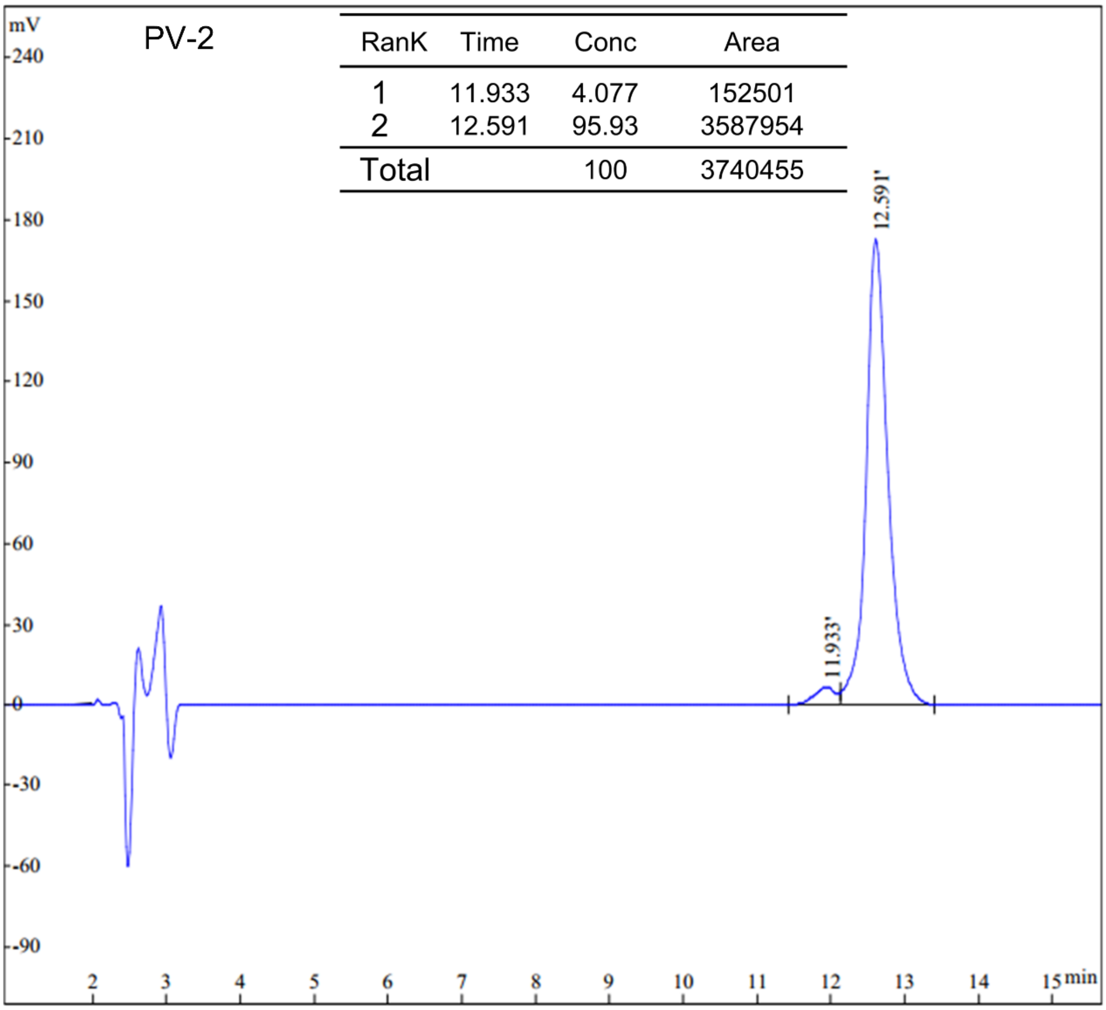
PV-2
RanK Time Conc Area
1 11.933 4.077 152501
2 12.591 95.93 3587954
Total 100 3740455
12.591'
11.933'
mV
min

(g)

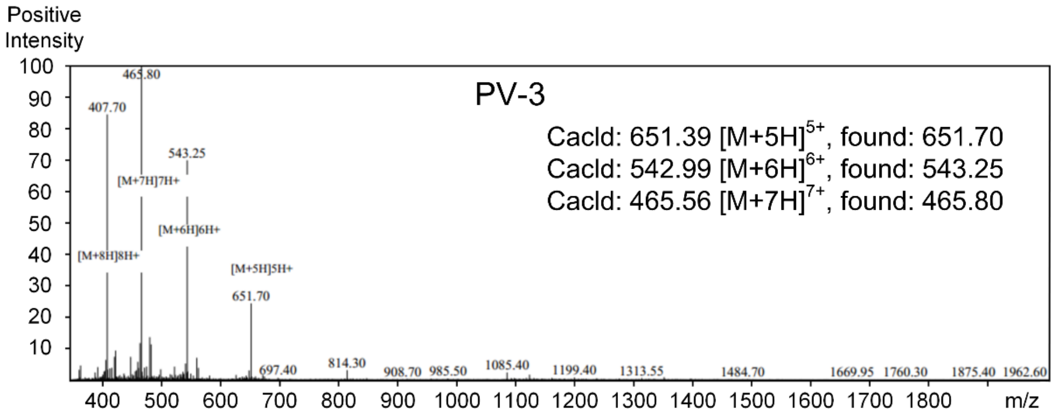
Positive Intensity
PV-3
Cacld: 651.39 [M+5H]5+, found: 651.70
Cacld: 542.99 [M+6H]6+, found: 543.25
Cacld: 465.56 [M+7H]7+, found: 465.80
465.80
407.70
543.25
[M+7H]7H+
[M+6H]6H+
[M+8H]8H+
[M+5H]5H+
651.70
m/z

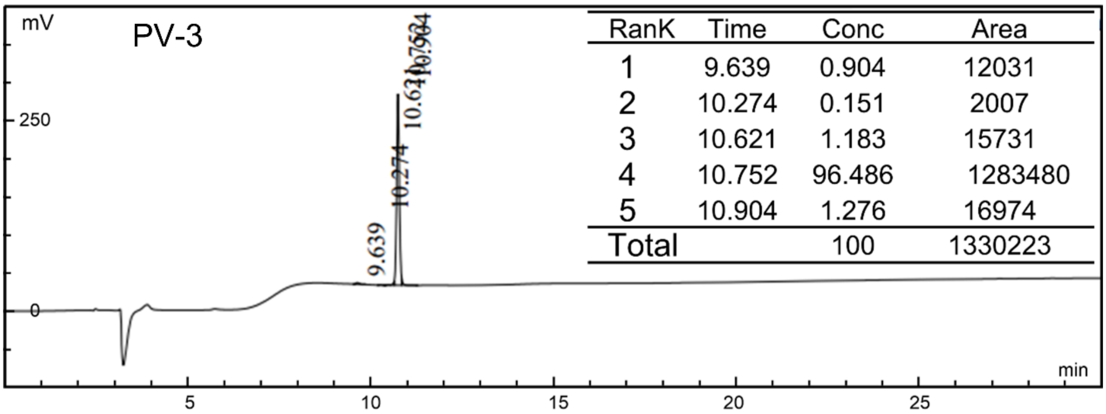

RanK	Time	Conc	Area
1	9.639	0.904	12031
2	10.274	0.151	2007
3	10.621	1.183	15731
4	10.752	96.486	1283480
5	10.904	1.276	16974
Total		100	1330223

(h)

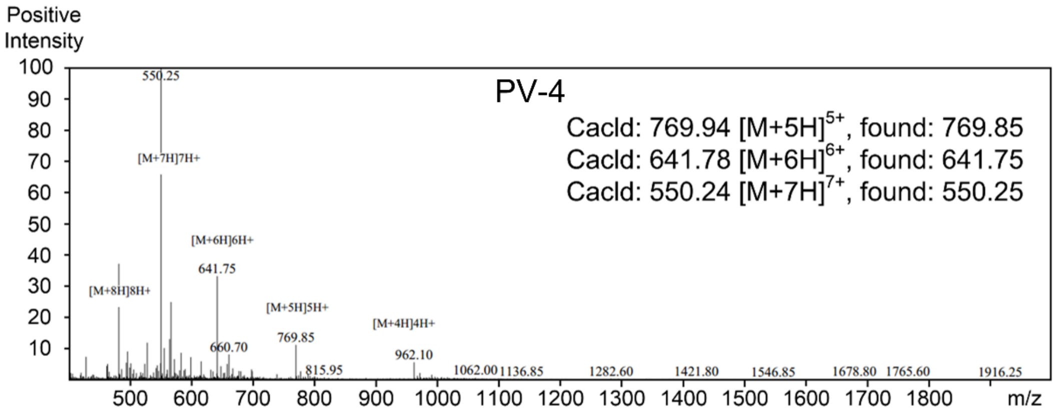

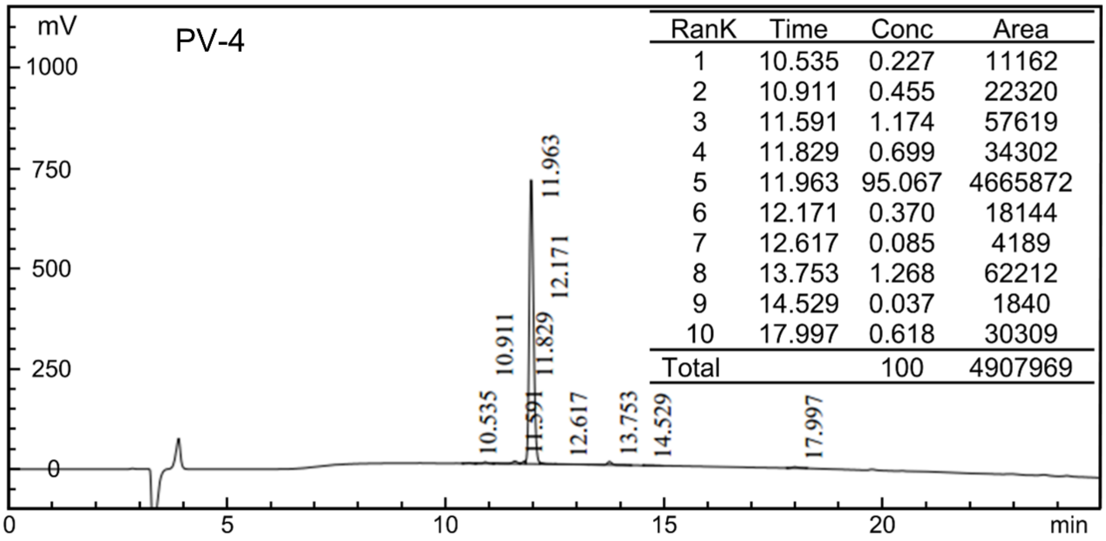

RanK	Time	Conc	Area
1	10.535	0.227	11162
2	10.911	0.455	22320
3	11.591	1.174	57619
4	11.829	0.699	34302
5	11.963	95.067	4665872
6	12.171	0.370	18144
7	12.617	0.085	4189
8	13.753	1.268	62212
9	14.529	0.037	1840
10	17.997	0.618	30309
Total		100	4907969

(**i**)

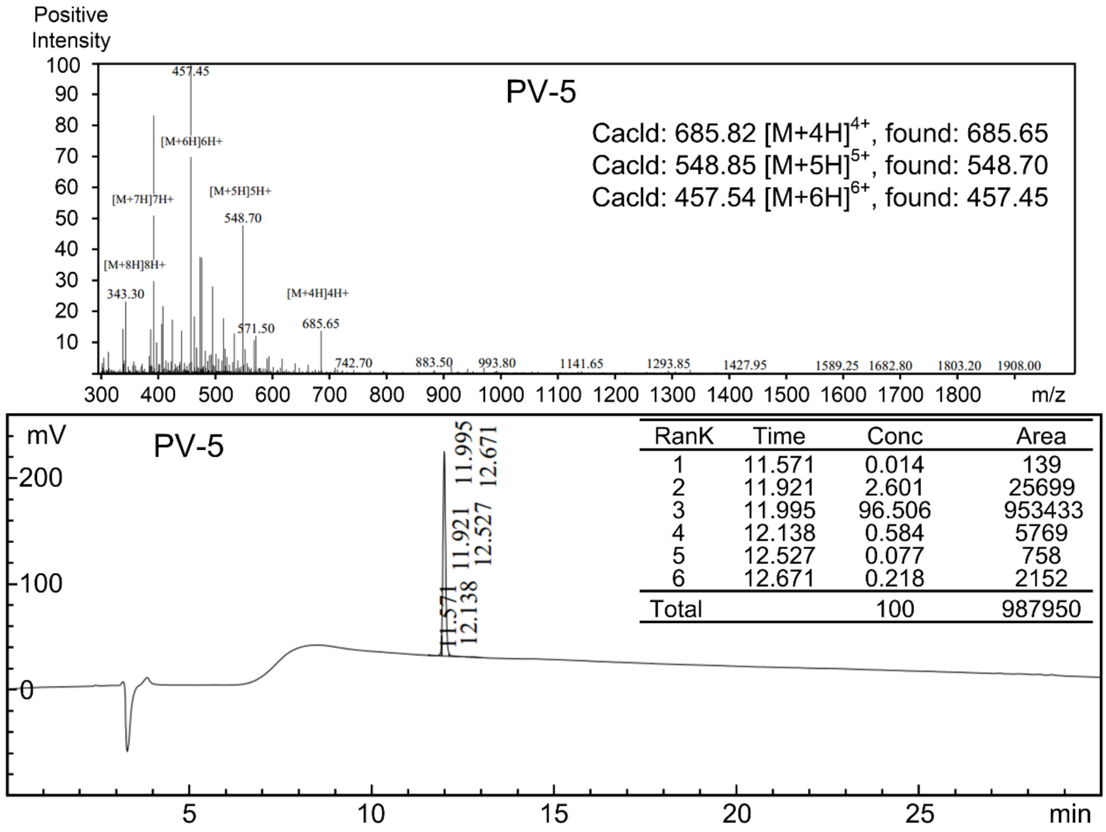

(**a**)-(**i**) ESI mass spectrum analysis and HPLC analysis of TP10, GALA, Penetratin, dfTAT, PV-1, PV-2, PV-3, PV-4 and PV-5, respectively.

Note S3. The synthesis procedures of probes for microtubules.

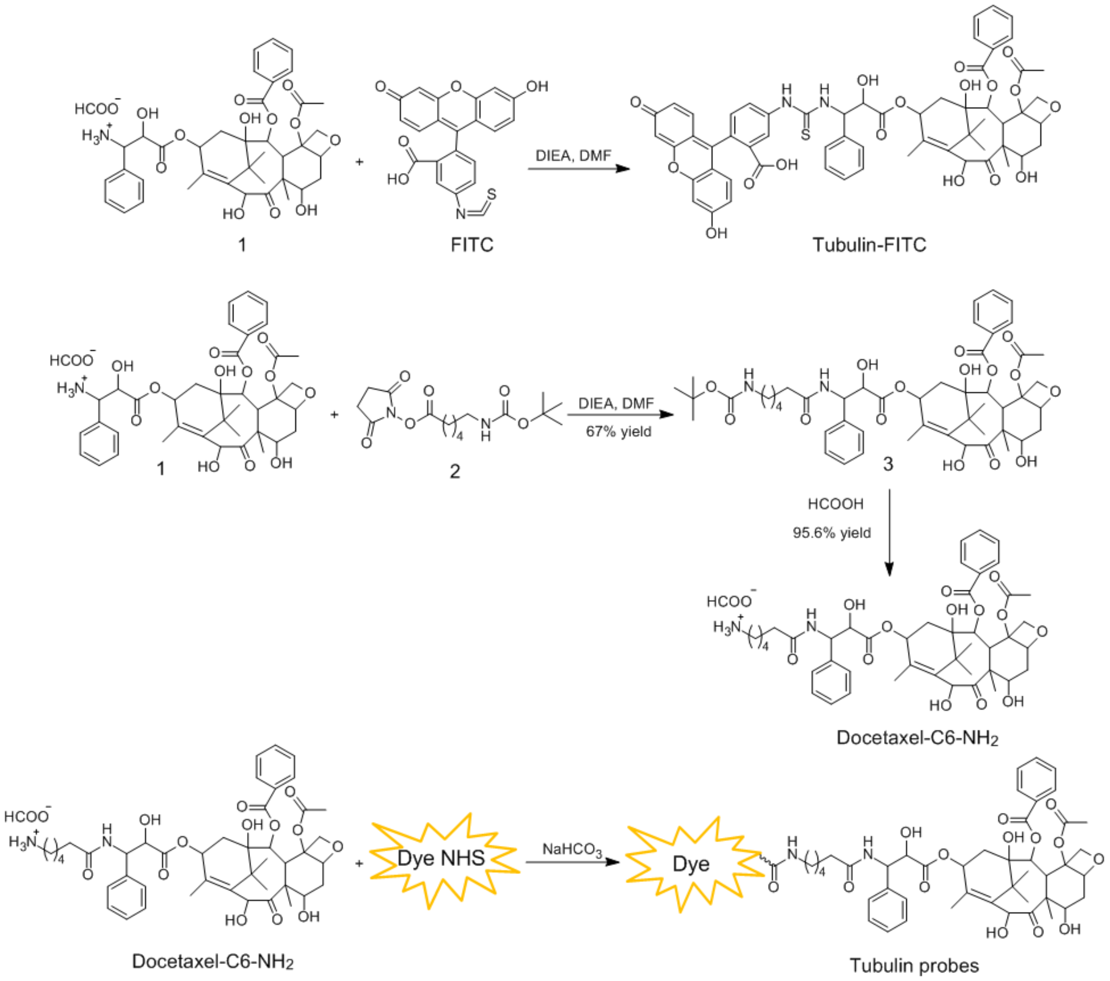

Preparation of Tubulin-FITC. 3'-aminodocetaxel **5** was synthesized as previously reported[36]. To a stirred solution of **5** (75.3 mg, 0.1 mmol) in dimethyl sulfoxide (DMSO, 2 mL), N,N-diisopropylethylamine (DIEA, 511 µL, 2.9 mmol) and fluorescein isothiocyanate (FITC, 19.5 mg, 0.05 mmol) were added. The mixture was kept stirring at room temperature overnight. Then the solvent was removed under reduced pressure, and the residue was purified by preparative high-performance liquid chromatography (HPLC, solvent A: 0.1% trifluoroacetic acid in 100% acetonitrile, solvent B: 0.1% trifluoroacetic acid in 100% water) to a purity of > 95% to give Tubulin-FITC, and its mass was confirmed by electrospray ionization mass spectrometry (ESI-MS) (**Note S4**).

Preparation of 7. Compound **6** was synthesized as previously reported[37]. To a solution of **5** (220 mg, 0.29 mmol) in dimethylformamide (DMF, 10 mL), compound **6** (191 mg, 0.58 mmol) and DIEA (152 µL, 0.87 mmol) were added. The mixture was kept stirring at room temperature overnight. Then the solvent was removed under reduced pressure, and the residue was purified by flash silica column chromatography (3% CH_3OH/CH_2Cl_2 to 10% CH_3OH/CH_2Cl_2) to give **7** as a white solid (180 mg, 0.20 mmol, 67% yield).

Preparation of Docetaxel-C6-NH2. Compound **7** (100 mg, 0.11mmol) was dissolved

in formic acid (HCOOH, 5 mL) and the mixture was kept stirring at room temperature for 30 min. Then the solvent was removed under reduced pressure. The residue was precipitated and washed with cold ether to give Docetaxel-C6-NH_2 (90.4 mg) as a white solid, which was used directly in next step without further purification. The HPLC analysis and ESI mass spectrum are shown in **Note S4**.

Preparation of Tubulin-Atto 488, Tubulin-Atto 514, Tubulin-Atto 565, Tubulin-Cy3B, Tubulin-Alexa 488, Tubulin-Alexa 647, and Tubulin-STAR 635P. The commercial available dyes Atto 488, Atto 514, Atto 565 (Sigma-Aldrich Co., LLC), Cy3B (GE Healthcare UK limited), or Alexa 488, Alexa 647 (Thermo Fisher Scientific, Inc.) containing an N-hydroxysuccinimidyl (NHS) moiety (33 nmol) were dissolved in DMF (10 μL). Then Docetaxel-C6-NH_2 (1 mM in phosphate buffered solution, pH 7.4, 100 μL) and $NaHCO_3$ (0.1 M in phosphate buffered solution, 100 μL) were added to the mixture. After kept shaking on a platform rocker overnight, the mixture was concentrated under reduced pressure and the crude product was used for live-cell labeling without further purification.

Note S4. Characterization of Tubulin-FITC and Docetaxel-C6-NH_2.

(a)

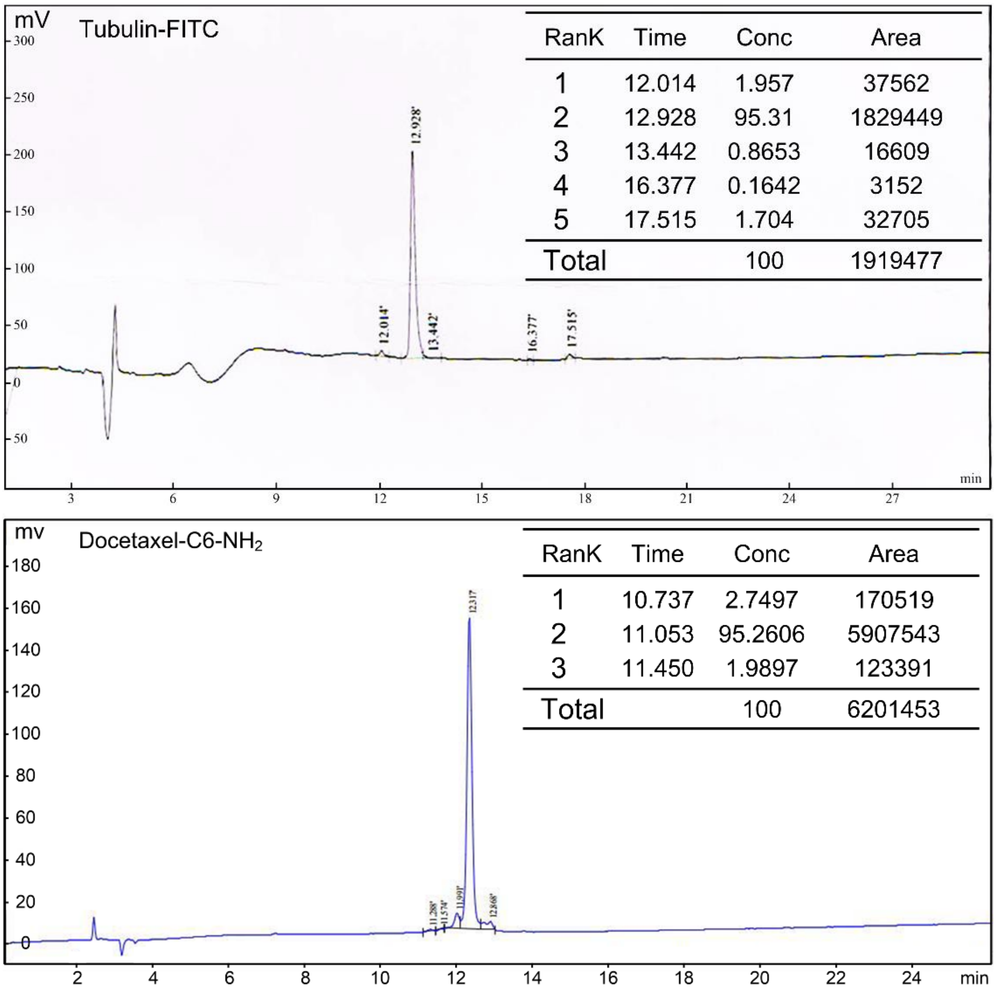

(b)

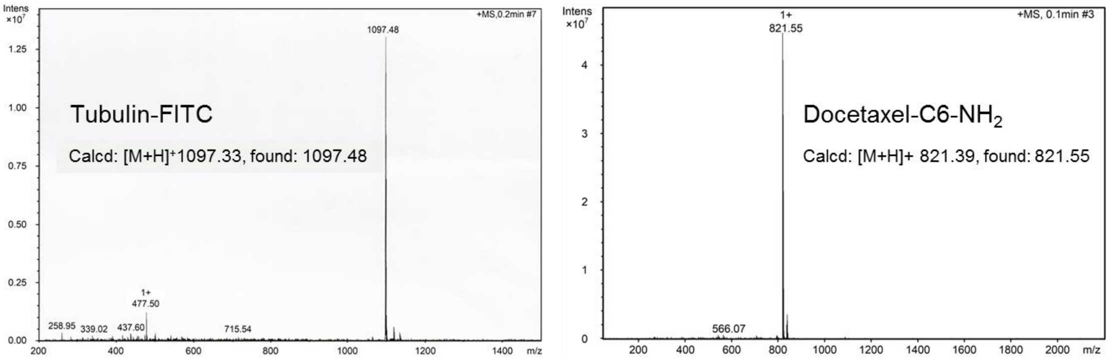

(**a**) HPLC analysis and (**b**) ESI mass spectrum analysis of Tubulin-FITC and Docetaxel-C6-NH_2.

Note S5. The synthesis procedures of Hoechst-C3-NH_2, Hoechst-Alexa 488, Hoechst-Alexa 647, Hoechst-Cy3B, and Hoechst-Atto 514.

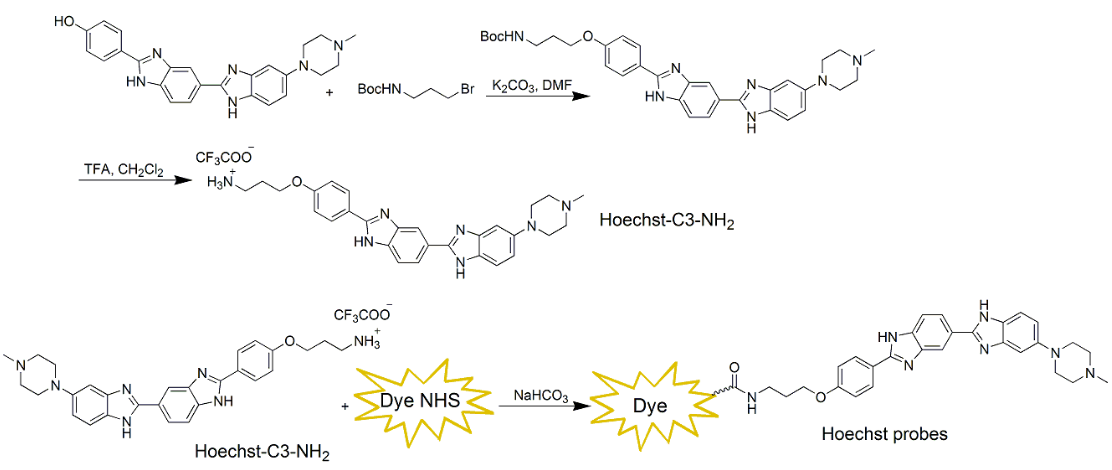

Preparation of Hoechst-C3-NH_2. Hoechst-C3-NH_2 was synthesized as previously reported[38] from commercial Hoechst 33258 hydrochloride. The product was purified by reverse-phase preparative HPLC (solvent A: 0.1% trifluoroacetic acid in 100% acetonitrile, solvent B: 0.1% trifluoroacetic acid in 100% water) to a purity of > 95%, and its mass was confirmed by ESI-MS (**Note S6**).

Preparation of Hoechst-Alexa 488, Hoechst-Alexa 647, Hoechst-Cy3B, and Hoechst-Atto 514. The commercial available dyes Alexa 488, Alexa 647 (Thermo Fisher Scientific, Inc.), Cy3B (GE Healthcare UK limited), or Atto 514 (Sigma-Aldrich Co., LLC) containing a NHS moiety (33 nmol) were dissolved in DMF (10 μL). Then Hoechst-C3-NH_2 (1 mM in phosphate buffered solution, pH 7.4, 100 μL) and $NaHCO_3$ (0.1 M in phosphate buffered solution, 100 μL) were added to the mixture. After kept shaking on a platform rocker overnight, the mixture was concentrated under reduced pressure and the crude product was used for live-cell labeling without further purification.

Note S6. Characterization of Hoechst-C3-NH_2.

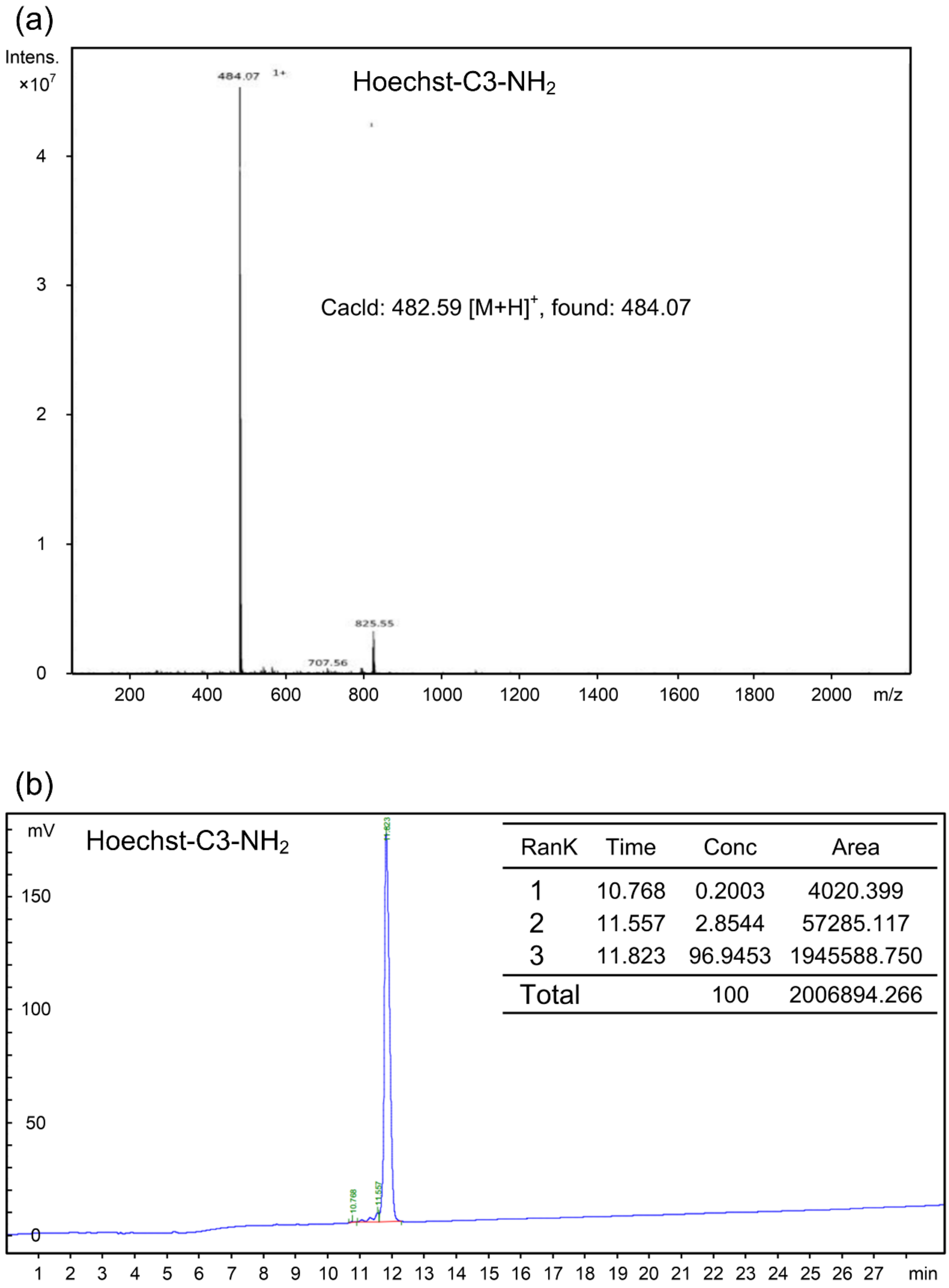

RanK	Time	Conc	Area
1	10.768	0.2003	4020.399
2	11.557	2.8544	57285.117
3	11.823	96.9453	1945588.750
Total		100	2006894.266

(**a**) ESI mass spectrum analysis and (**b**) HPLC analysis of Hoechst-C3-NH_2

Note S7. The synthesis procedures of Morph-Alexa 488, Morph-Alexa 647, Morph-Cy3B, Morph-Atto 514, and Morph-Atto 488.

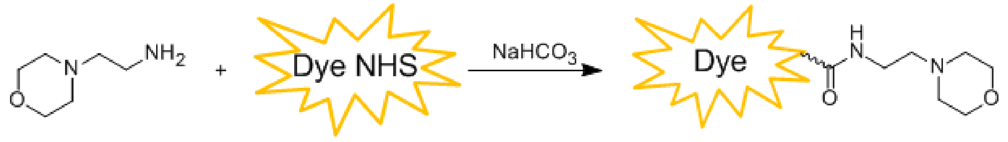

Preparation of Morph-Alexa 488, Morph-Alexa 647, Morph-Atto 488, Morph-Atto 514, and Morph-Cy3B. The commercial available dyes Alexa 488, Alexa 647 (Thermo Fisher Scientific, Inc.), Atto 488, Atto 514 (Sigma-Aldrich Co., LLC), or Cy3B (GE Healthcare UK limited) containing a NHS moiety (33 nmol) were dissolved in DMF (10 μL). Then commercial available Morph-C2-NH_2 (1 mM in PBS, pH 7.4, 100 μL) and $NaHCO_3$ (0.1 M in PBS, 100 μL) were added to the mixture. After kept stirring overnight, the mixture was concentrated under reduced pressure and the crude product was used for live-cell labeling without further purification.

References

36. Lukinavičius, G. *et al.* Fluorogenic probes for live-cell imaging of the cytoskeleton., *Nature Methods* **11**, 731-733 (2014).
37. Tsuboi, K. *et al.* Potent and selective inhibitors of glutathione S-transferase omega 1 that impair cancer drug resistance. *Journal of the American Chemical Society* **133**, 16605-16616 (2011).
38. Lukinavičius, G. *et al.* SiR–Hoechst is a far-red DNA stain for live-cell nanoscopy. *Nature Communications* **6**, 8497–8503 (2015).
